# Trends in Diabetic Medication Use and Hypoglycemia Incidence Among Older Adults in Japan: A Retrospective Observational Study and a 10-Year Analysis Based on Level of Care Need Using Linked Medical and Long-Term Care Data

**DOI:** 10.7759/cureus.87486

**Published:** 2025-07-07

**Authors:** Yasuhiro Niida, Yuichi Nishioka, Tomoya Myojin, Tatsuya Noda, Yutaka Takahashi, Tomoaki Imamura

**Affiliations:** 1 Department of Public Health, Health Management and Policy, Nara Medical University, Kashihara, JPN; 2 Department of Diabetes and Endocrinology, Nara Medical University, Kashihara, JPN

**Keywords:** diabetes, frailty, hypoglycemia, insulin, older adults, sulfonylurea

## Abstract

Background

In Japan, the prevalence of diabetes among those aged 75 years and older is increasing with an aging population. The main goal of diabetes care is to prevent complications (such as diabetic nephropathy, retinopathy, neuropathy, and arteriosclerotic disease) while maintaining the quality of life. However, in older adults, glycemic targets must be individualized to avoid hypoglycemia, especially with medications, including sulfonylureas (SUs), glinides (GLs), and insulin, which pose a high risk of hypoglycemia. This study aimed to investigate the prescription trends of diabetes medications and the incidence of hypoglycemia among older diabetic patients aged 75 and above in Japan, stratified by their level of care need. This study analyzed 22,070-38,001 outpatients aged ≥75 years, assessing medication trends and hypoglycemia incidence (0.594% in 2013 to 0.316% in 2022).

Methods

This was a retrospective, observational study. Data were obtained from the Kokuho Database in Nara Prefecture (Nara KDB), linking medical and long-term care insurance records. This study included outpatients aged 75 and above with diabetes (excluding type 1) from 2013 to 2022. Patients were grouped as having care needs (HCN) or no care needs (NCN). The proportion of prescriptions for diabetes medications and hypoglycemia requiring intravenous glucose was annually assessed.

Results

From 2013 to 2022, the proportion of prescriptions for sulfonylureas (from 40.6% (N = 8,958) to 18.7% (N = 7,113)), intermediate-acting insulins (IIs) (from 8.17% (N = 1,804) to 1.79% (N = 681)), and rapid-acting insulins (RIs) (from 10.6% (N = 2,334) to 6.38% (N =2,424)) declined, similar to the incidence of hypoglycemia (from 0.594% (N = 131) to 0.316% (N = 120)). The incidence of hypoglycemia declined overall (0.594% to 0.316%), but that in HCN rose to 0.770% in 2022. The decrease in the incidence of hypoglycemia between 2013 and 2022 was statistically significant (p < 0.001). The number of long-acting insulin (LI) (from 5.66% (N = 1,250) to 8.13% (N = 3,091)) and glinide (from 4.78% (N = 1,055) to 5.87% (N = 2,231)) prescriptions increased. Dipeptidyl peptidase-4 inhibitors (DPP4Is) (from 56.8% (N = 12,540) to 72.6% (N = 27,573)) were the most commonly prescribed drugs. HCN consistently showed a higher proportion of hypoglycemia (from 1.25% (N = 54) to 0.824% (N = 43)) than NCN (from 0.434% (N = 77) to 0.235% (N = 77)).

Conclusion

Diabetes management in older patients in Japan has improved, with a continuing decline in the incidence of hypoglycemia. However, older adults with higher care needs remain more vulnerable to hypoglycemia, likely due to greater frailty and higher insulin use. This finding suggests the need for further individualized treatment strategies and close monitoring.

## Introduction

In 2021, there were 529 million people with diabetes worldwide; the global age-adjusted total prevalence of diabetes was 6.1% [1]. In Japan, the age-adjusted prevalence of people with highly suspected diabetes (HbA1c ≥ 6.5% or receiving diabetes treatment) in 2023 was 11.4% for men and 6.4% for women [2]. With an aging population, the number of older adults with diabetes has increased. The goal of diabetes management is to prevent the onset and progression of vascular complications and achieve a life expectancy and quality of life equivalent to those of people without diabetes through the prevention and management of complications and advocacy. To this end, appropriate blood glucose levels, blood pressure, serum lipid levels, and weight management are required [3,4]. General targets for HbA1c have been established [3].

However, Japanese guidelines recommend individualized glycemic control targets in older adults with diabetes to avoid the occurrence of hypoglycemia [5]. Sulfonylureas (SUs), glinides (GLs), and insulins, known to be associated with a high risk of hypoglycemia even when used alone, should be used cautiously [5]. A similar view has been expressed in the "Managing Older People With Type 2 Diabetes: Global Guidelines" published by the International Diabetes Federation in 2013 [6], suggesting an international consensus. In spite of the publication of this guideline, around the world, glycemic control targets for people with diabetes who need care may not be individualized and may remain strict [7-10].

The primary aim of the present study was to investigate the prescription status of diabetes medicines (in particular, sulfonylureas, glinides, and insulins, which pose a high risk of hypoglycemia when used alone) and the incidence of hypoglycemia in diabetic patients aged 75 years and above by level of care need over time. The secondary aim was to describe the transition of the diabetes management status of older diabetic patients with and without a high level of care need.

## Materials and methods

This study was approved by the Ethics Committee of Nara Medical University (approval number: 1123, date: September 18, 2024). The need for informed consent was waived because this study was based on a retrospective analysis of already anonymized, routinely collected data that do not allow for the identification of specific individuals and for which a correspondence table has not been created.

Database

The Kokuho Database by the Federation of National Health Insurance Organizations in Nara Prefecture (Nara KDB), in central Japan, was used to source data. The population of Nara Prefecture was approximately 1.32 million in the 2020 census, of which 215,000 were aged 75 years and above. The population of Japan in the same year was approximately 126 million, indicating that Nara Prefecture accounted for approximately 1% of Japan's population as a whole. Nara KDB includes the monthly claims data of health insurance for National Health Insurance (NHI) (age < 75 years) and Later-Stage Elderly Medical Care System (LSEMCS) (age ≥ 75 years); it also includes the results of daily care-service data from long-term care insurance (LTCI) for NHI and LSEMCS subscribers. Almost all people aged 75 and above are enrolled in the LSEMCS. All data were linked using an identifier in the database. Subscriber lists included age, sex, postal code, observation period, reason for withdrawal, and date of death during the study period. The available information from the health insurance data included the patient's disease, treatment, care provided to both outpatients and hospitalized patients, and the corresponding costs. The care insurance data included support and care level, along with information about the care services provided for insured individuals. Disease names were based on International Classification of Diseases, Tenth Revision (ICD-10) codes and included Japanese disease codes corresponding to ICD-10 codes. The care level (non-certified, Support 1, Support 2, Care 1, Care 2, Care 3, Care 4, and Care 5) was recorded according to the LTCI for the care data. Nara KDB, used widely in previous studies, was used for research purposes [11,12]. The Japanese disease, ICD-10, and drug codes used in this study are shown in the Appendices.

Subjects

In this study, the data were analyzed by fiscal year from April 1, 2013, to March 31, 2023. The fiscal year in Japan is from April to March of the following year. For example, the fiscal year 2013 represents the period from April 1, 2013, to March 31, 2014. That is to say, the observation period of this study was from the fiscal year 2013 to 2022. Outpatients with diabetes, excluding type 1 diabetes, aged 75 years or older at the start of each year were included. For each subject, the care level and details of the diabetic medication prescribed at the end of each year and the incidence of hypoglycemia during the period were tabulated. Diabetes (excluding type 1 diabetes) was defined using disease and drug codes. Hypoglycemia was defined as the presence of disease codes and drug codes for 50% or 70% glucose solutions on the same day. That is, in this study, hypoglycemia was defined as low blood glucose and an inability to take glucose orally, requiring intravenous injections in a medical facility. Care 2-5 were defined as having care needs (HCN, loss of activities of daily living (ADLs)), and the others as having no care needs (NCN, no loss of ADL). A previous study in Japan using care data from the LTCI defined Care 2-5 as a loss of healthy life expectancy (loss of ADL) [13].

Definition of diabetes, excluding patients with type 1 diabetes

We defined patients with diabetes as those who had a disease code of diabetes and were prescribed diabetes medication, excluding those who had a disease code of type 1 diabetes and were prescribed insulin with a self-injection fee for type 1 diabetes. This definition referred to the previous studies [14,15]. A list of disease and drug codes used is provided in the Appendices.

Definition of hypoglycemia

According to the previous study [16], we defined patients with hypoglycemia as those who had a disease code of hypoglycemia and were prescribed intravenous injections of 50% or 70% glucose on the same day. A list of the disease and drug codes used is provided in the Appendices.

Outcome

The main outcome of this study was the description of diabetic drug prescriptions (α-glucosidase inhibitors (αGIs), biguanides (BGs), dipeptidyl peptidase-4 inhibitors (DPP4Is), glinides (GLs), glucagon-like peptide-1 receptor agonists (GLP1RAs), sodium-coupled glucose transporter 2 inhibitors (SGLT2Is), sulfonylureas (SUs), thiazolidines (TZDs), intermediate-acting insulins (IIs), long-acting insulins (LIs), and rapid-acting insulins (RIs)) and incidence of hypoglycemia in outpatients with diabetes excluding those with type 1 diabetes and patients aged 75 years and above, by year. Changes over time were described and stratified into HCN and NCN groups.

Variables

The demographic data included age and sex. Medical data included hypertension and dyslipidemia. Both were defined using disease and drug codes. A list of disease and drug codes used is provided in the Appendices.

Analysis

We used Microsoft SQL Server (version 18.4) (Microsoft Corp., Redmond, WA) for data management and Microsoft Excel (version 365) for data analysis (counting, calculation, and test). The rate of change in the proportion of prescriptions for diabetic drugs and the incidence of hypoglycemia were calculated using the least squares method. The rate of change was calculated separately for the periods from 2013 to 2017 and from 2017 to 2022. The least squares method was used simply to calculate the rate of change. The Chi-square test was used to evaluate statistical significance. We considered a P value < 0.05 to be statistically significant.

## Results

**Figure 1:**
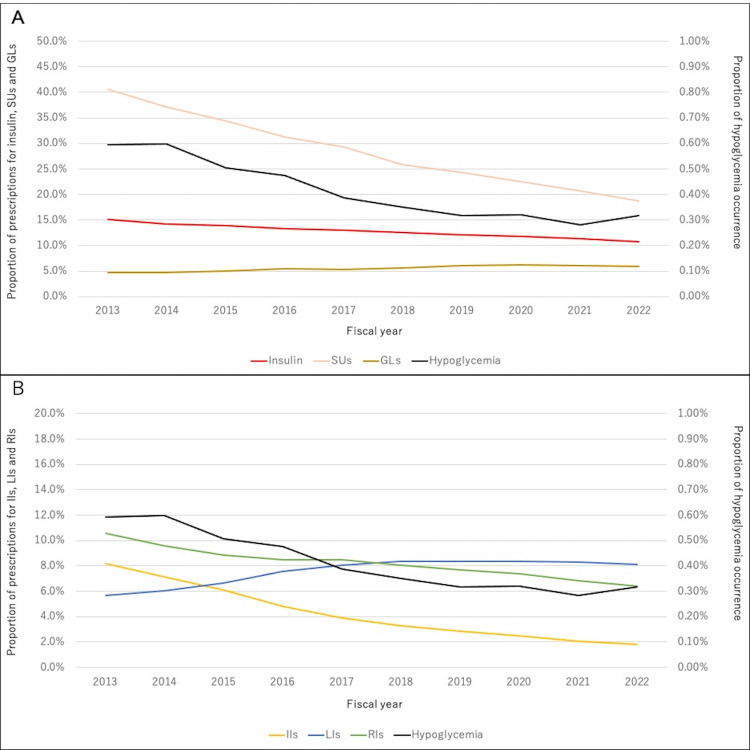
Changes in the proportion of hypoglycemia occurrence and prescriptions A: Changes in the proportion of hypoglycemia occurrence and prescriptions for all insulins, SUs, and GLs over time. The black line indicates hypoglycemia, the red line indicates insulins, the pink line indicates SUs, and the gold line indicates GLs. B: Changes in the proportion of hypoglycemia occurrence and prescriptions for IIs, LIs, and RIs over time. The black line indicates hypoglycemia, the yellow line indicates IIs, the blue line indicates LIs, and the green line indicates RIs. SUs: sulfonylureas, GLs: glinides, IIs: intermediate-acting insulins, LIs: long-acting insulins, RIs: rapid-acting insulins

**Figure 2:**
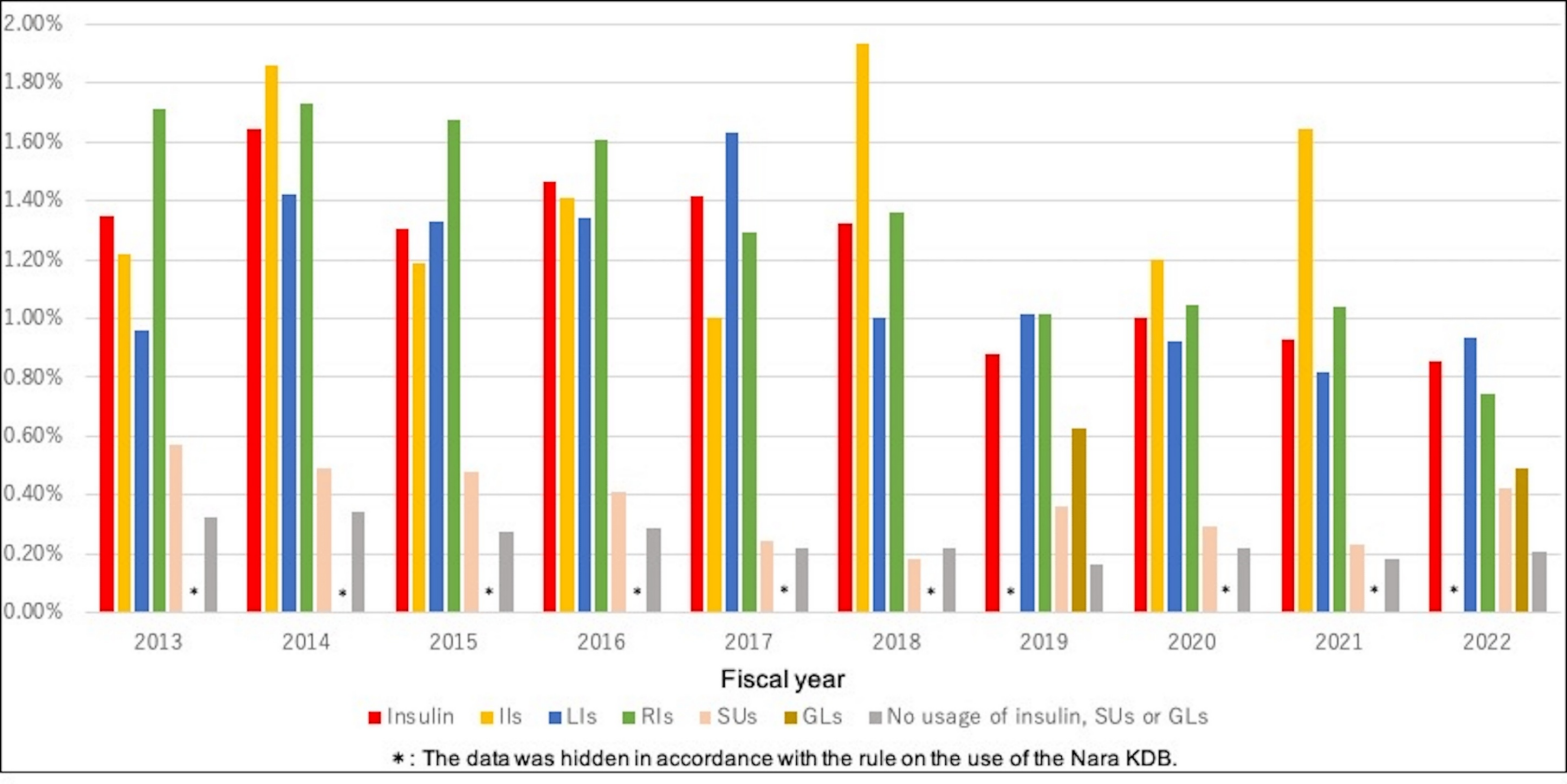
Proportion of hypoglycemia occurrence in people using insulin, SUs, GLs, and other diabetes drugs by year The black line indicates hypoglycemia, the red line indicates insulins, the pink line indicates SUs, the gold line indicates GLs, the yellow line indicates IIs, the blue line indicates LIs, and the green line indicates RIs. SUs: sulfonylureas, GLs: glinides, IIs: intermediate-acting insulins, LIs: long-acting insulins, RIs: rapid-acting insulins

Table 1 shows the characteristics of the participants by year. The number of participants increased annually. The proportion of men among the participants was approximately 50%, and this proportion increased annually. Changes in the age structure of participants were observed, with an increasing proportion of older adults. The proportion of prescriptions for insulin, IIs, RIs, and SUs decreased between 2013 and 2022, similar to the incidence of hypoglycemia (Figure 1A, 1B). The decrease in the incidence of hypoglycemia between 2013 and 2022 was statistically significant (p < 0.001). The proportion of prescriptions for LIs and GLs increased (Figure 1A, 1B). The proportion of prescriptions for GIs and TZDs decreased, whereas those for BGs, DPP4Is, GLP1RAs, and SGLT2Is increased. DPP4Is were the most frequently prescribed drugs during the study period. There was no change in the proportion of patients with hypertension, but that of patients with dyslipidemia increased. Figure 2 shows the proportion of hypoglycemia occurrence in people using insulin, SUs, GLs, and other diabetes drugs by year. Users of insulin, SUs, and GLs had a higher rate of hypoglycemia than users of other diabetes drugs. These trends remained unchanged after adjusting for age (Table 2).

**Table 1:** Characteristics of the subjects and proportion of drug prescriptions and hypoglycemia occurrence by year

Fiscal year	2013	2014	2015	2016	2017	2018	2019	2020	2021	2022
Total subjects (number)	22070	23420	24923	26522	28138	29943	31950	33588	35129	38001
Male (number (%))	11043 (50.0)	11814 (50.4)	12729 (51.1)	13694 (51.6)	14653 (52.1)	15668 (52.3)	16822 (52.7)	17737 (52.8)	18581 (52.9)	20089 (52.9)
Mean age (years)	81.2	81.4	81.5	81.5	81.5	81.6	81.6	81.8	82.1	82.2
Age 75-79 (number (%))	10574 (47.9)	10977 (46.9)	11557 (46.4)	12081 (45.6)	12833 (45.6)	13288 (44.4)	14479 (45.3)	14914 (44.4)	14505 (41.3)	15139 (39.8)
Age 80-84 (number (%))	6855 (31.1)	7328 (31.3)	7807 (31.3)	8424 (31.8)	8907 (31.7)	9694 (32.4)	9975 (31.2)	10555 (31.4)	11413 (32.5)	12537 (33.0)
Age 85-89 (number (%))	3446 (15.6)	3750 (16.0)	4065 (16.3)	4316 (16.3)	4576 (16.3)	4953 (16.5)	5273 (16.5)	5663 (16.9)	6486 (18.5)	7168 (18.9)
Age 90-94 (number (%))	1012 (4.59)	1153 (4.92)	1224 (4.91)	1411 (5.32)	1512 (5.37)	1694 (5.66)	1860 (5.82)	2054 (6.12)	2239 (6.37)	2565 (6.75)
Age ≥ 95 (number (%))	183 (0.829)	212 (0.905)	270 (1.08)	290 (1.09)	310 (1.10)	314 (1.05)	363 (1.14)	402 (1.20)	486 (1.38)	592 (1.56)
Hypoglycemia (number (%))	131 (0.594)	140 (0.598)	126 (0.506)	126 (0.475)	109 (0.387)	105 (0.351)	101 (0.316)	108 (0.322)	99 (0.282)	120 (0.316)
Insulin (all) (number (%))	3336 (15.1)	3344 (14.3)	3449 (13.8)	3544 (13.4)	3666 (13.0)	3772 (12.6)	3860 (12.1)	3976 (11.8)	3971 (11.3)	4089 (10.8)
Insulin (intermediate-acting) (number (%))	1804 (8.17)	1666 (7.11)	1516 (6.08)	1277 (4.81)	1097 (3.90)	982 (3.28)	915 (2.86)	833 (2.48)	729 (2.08)	681 (1.79)
Insulin (long-acting) (number (%))	1250 (5.66)	1407 (6.01)	1656 (6.64)	2012 (7.59)	2266 (8.05)	2497 (8.34)	2662 (8.33)	2811 (8.37)	2922 (8.32)	3091 (8.13)
Insulin (rapid-acting) (number (%))	2334 (10.6)	2250 (9.61)	2207 (8.86)	2242 (8.45)	2394 (8.51)	2420 (8.08)	2454 (7.68)	2485 (7.40)	2406 (6.85)	2424 (6.38)
Sulfonylurea (number (%))	8958 (40.6)	8696 (37.1)	8575 (34.4)	8291 (31.3)	8231 (29.3)	7726 (25.8)	7753 (24.3)	7539 (22.4)	7254 (20.6)	7113 (18.7)
Glinide (number (%))	1055 (4.78)	1095 (4.68)	1262 (5.06)	1435 (5.41)	1476 (5.25)	1695 (5.66)	1915 (5.99)	2086 (6.21)	2129 (6.06)	2231 (5.87)
α-Glucosidase inhibitor (number (%))	5127 (23.2)	5033 (21.5)	5079 (20.4)	5097 (19.2)	5060 (18.0)	4838 (16.2)	5057 (15.8)	5023 (15.0)	4858 (13.8)	4727 (12.4)
Biguanide (number (%))	3090 (14.0)	3424 (14.6)	4018 (16.1)	4664 (17.6)	5514 (19.6)	6559 (21.9)	7756 (24.3)	8806 (26.2)	9573 (27.3)	10475 (27.6)
Dipeptidyl peptidase-4 inhibitor (number (%))	12540 (56.8)	15111 (64.5)	17307 (69.4)	19162 (72.2)	20999 (74.6)	22649 (75.6)	24565 (76.9)	25737 (76.6)	26414 (75.2)	27573 (72.6)
Glucagon-like peptide-1 receptor agonist (number (%))	39 (0.177)	61 (0.260)	101 (0.405)	224 (0.845)	346 (1.23)	522 (1.74)	698 (2.18)	978 (2.91)	1329 (3.78)	1652 (4.35)
Sodium-coupled glucose transporter 2 inhibitor (number (%))	0 (0)	94 (0.401)	393 (1.58)	855 (3.22)	1616 (5.74)	2543 (8.49)	4323 (13.5)	5920 (17.6)	8474 (24.1)	11786 (31.0)
Thiazolidines (number (%))	2508 (11.4)	2435 (10.4)	2470 (9.91)	2443 (9.21)	2470 (8.78)	2476 (8.27)	2465 (7.72)	2471 (7.36)	2358 (6.71)	2355 (6.20)
Hypertension (number (%))	17066 (77.3)	18023 (77.0)	19078 (76.5)	20180 (76.1)	21477 (76.3)	22777 (76.1)	24290 (76.0)	25540 (76.0)	26972 (76.8)	29447 (77.5)
Dyslipidemia (number (%))	10702 (48.5)	11625 (49.6)	12695 (50.9)	13706 (51.7)	14742 (52.4)	16078 (53.7)	17525 (54.9)	18865 (56.2)	20286 (57.7)	22486 (59.2)

**Table 2:** Age-adjusted proportion of drug prescriptions and hypoglycemia occurrence by year

Fiscal year	2013	2014	2015	2016	2017	2018	2019	2020	2021	2022
Hypoglycemia (%)	0.665	0.701	0.553	0.526	0.414	0.362	0.346	0.333	0.299	0.328
Insulin (all) (%)	14.8	14.1	13.6	13.1	12.8	12.4	11.9	11.7	11.2	10.6
Insulin (intermediate-acting) (%)	8.01	7.00	5.99	4.74	3.86	3.25	2.82	2.46	2.05	1.77
Insulin (long-acting) (%)	5.48	5.88	6.52	7.39	7.85	8.19	8.23	8.28	8.21	8.03
Insulin (rapid-acting) (%)	10.2	9.33	8.60	8.18	8.25	7.84	7.43	7.23	6.70	6.26
Sulfonylurea (%)	40.7	37.1	34.4	31.2	29.3	25.7	24.2	22.4	20.6	18.6
Glinide (%)	4.71	4.57	4.93	5.26	5.11	5.49	5.84	6.08	5.97	5.83

**Figure 3:**
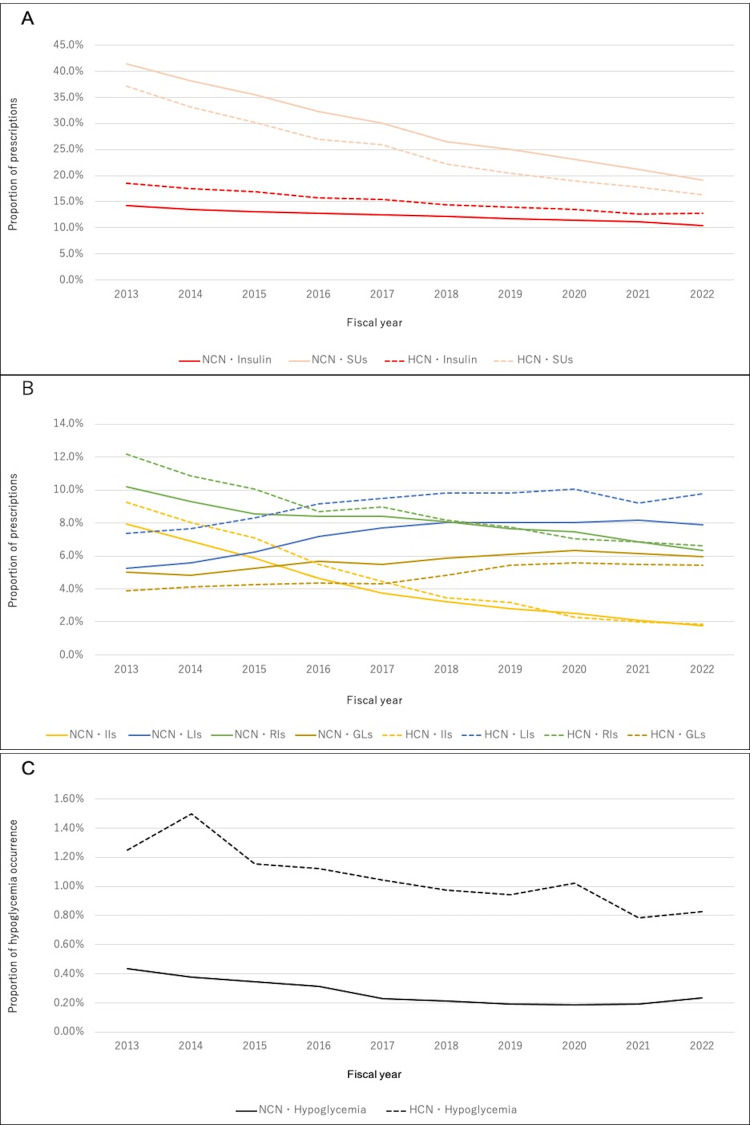
Changes in the proportion of prescriptions for diabetes and hypoglycemia occurrence over time in HCN and NCN A: Changes in the proportion of prescriptions for all insulins and SUs over time in the HCN and NCN. The red line indicates insulins, and the pink line indicates SUs. B: Changes in the proportion of prescriptions for IIs, LIs, RIs, and GLs over time in HCN and NCN. The yellow line indicates IIs, the blue line indicates LIs, the green line indicates RIs, and the gold line indicates GLs. C: Changes in the proportion of hypoglycemia occurrence over time in HCN and NCN. The dotted line indicates HCN, and the solid line indicates NCN. SUs: sulfonylureas, GLs: glinides, IIs: intermediate-acting insulins, LIs: long-acting insulins, RIs: rapid-acting insulins, HCN: having care needs group, NCN: no care needs group

Table 3 and Table 4, respectively, show the characteristics of NCN and HCN by year. The number of participants in both groups increased annually. In both groups, the age structure of the target population changed, with an increase in the proportion of older adults. The mean age of patients with HCN was higher than those with NCN. HCN had a higher proportion of older adults than NCN. NCN had a significantly higher proportion of men and dyslipidemia than HCN (p < 0.001 for both). On the other hand, there was no difference in the prevalence of hypertension. The proportion of prescriptions for insulin, IIs, RIs, and SUs decreased between 2013 and 2022, similar to the incidence of hypoglycemia (Figure 3A-3C). The proportion of prescriptions for LIs and GLs increased (Figure 3B). In both groups, the rate of change in the proportion of II, RI, and SU prescriptions and the incidence of hypoglycemia differed from 2017. Regarding the proportion of prescriptions for IIs, RIs, and SUs, the rate of decrease between 2017 and 2022 was slower than that between 2013 and 2017 (NCN: IIs, -1.05%/year versus -0.389%/year; RIs, -0.453%/year versus -0.402%/year; SUs, -2.84%/year versus -2.10%/year; HCN: IIs, -1.21%/year versus-0.520%/year; RIs, -0.857%/year versus -0.472%/year; SUs, -2.86%/year versus -1.80%/year). In terms of the incidence of hypoglycemia, the rate of decrease between 2017 and 2022 was slower than the rate of decrease between 2013 and 2017 in both groups (NCN: -0.0472%/year versus -0.00103%/year, HCN: -0.0800%/year versus -0.0446%/year). The proportion of prescriptions for αGIs and TZDs decreased, while that for BGs, DPP4Is, GLP1RAs, and SGLT2Is increased. DPP4Is were the most frequently prescribed drugs during the study period. There was no change in the proportion of patients with hypertension, but that of patients with dyslipidemia increased. These trends remained the same after adjusting for age (Tables 5, 6), but in terms of the incidence of hypoglycemia in HCN, the rate of decrease between 2017 and 2022 was higher compared to the rate of decrease between 2013 and 2017 (-0.0440%/year versus -0.0732%/year).

**Table 3:** Characteristics of NCN subjects and the proportion of drug prescriptions and hypoglycemia occurrence by year NCN: no care needs group

Fiscal year	2013	2014	2015	2016	2017	2018	2019	2020	2021	2022
Total subjects (number)	17739	18816	19974	21261	22662	24596	26633	28215	29914	32784
Male (number (%))	9314 (52.5)	9975 (53.0)	10698 (53.6)	11505 (54.1)	12373 (54.6)	13480 (54.8)	14675 (55.1)	15535 (55.1)	16508 (55.2)	18073 (55.1)
Mean age (years)	80.4	80.6	80.6	80.7	80.6	80.8	80.9	81.1	81.4	81.5
Age 75-79 (number (%))	9489 (53.5)	9880 (52.5)	10413 (52.1)	10891 (51.2)	11597 (51.2)	12147 (49.4)	13278 (49.9)	13766 (48.8)	13548 (45.3)	14358 (43.8)
Age 80-84 (number (%))	5535 (31.2)	5932 (31.5)	6289 (31.5)	6856 (32.2)	7298 (32.2)	8145 (33.1)	8491 (31.9)	8999 (31.9)	9981 (33.4)	11154 (34.0)
Age 85-89 (number (%))	2224 (12.5)	2440 (13.0)	2677 (13.4)	2840 (13.4)	3047 (13.4)	3406 (13.8)	3772 (14.2)	4170 (14.8)	4858 (16.2)	5480 (16.7)
Age 90-94 (number (%))	454 (2.56)	511 (2.72)	537 (2.69)	596 (2.80)	656 (2.89)	824 (3.35)	991 (3.72)	1150 (4.08)	1354 (4.53)	1554 (4.74)
Age ≥ 95 (number (%))	37 (0.209)	53 (0.282)	58 (0.290)	78 (0.367)	64 (0.282)	74 (0.301)	101 (0.379)	130 (0.461)	173 (0.578)	238 (0.726)
Hypoglycemia (number (%))	77 (0.434)	71 (0.377)	69 (0.345)	67 (0.315)	52 (0.229)	53 (0.215)	51 (0.191)	53 (0.188)	58 (0.194)	77 (0.235)
Insulin (all) (number (%))	2531 (14.3)	2540 (13.5)	2612 (13.1)	2718 (12.8)	2825 (12.5)	3004 (12.2)	3119 (11.7)	3248 (11.5)	3312 (11.1)	3425 (10.4)
Insulin (intermediate-acting) (number (%))	1404 (7.91)	1297 (6.89)	1166 (5.84)	989 (4.65)	853 (3.76)	797 (3.24)	746 (2.80)	710 (2.52)	625 (2.09)	583 (1.78)
Insulin (long-acting) (number (%))	930 (5.24)	1054 (5.60)	1244 (6.23)	1531 (7.20)	1747 (7.71)	1973 (8.02)	2141 (8.04)	2271 (8.05)	2443 (8.17)	2582 (7.88)
Insulin (rapid-acting) (number (%))	1806 (10.2)	1751 (9.31)	1709 (8.56)	1786 (8.40)	1902 (8.39)	1983 (8.06)	2041 (7.66)	2108 (7.47)	2048 (6.85)	2079 (6.34)
Sulfonylurea (number (%))	7352 (41.4)	7168 (38.1)	7080 (35.4)	6870 (32.3)	6812 (30.1)	6536 (26.6)	6663 (25.0)	6518 (23.1)	6324 (21.1)	6264 (19.1)
Glinide (number (%))	887 (5.00)	905 (4.81)	1052 (5.27)	1207 (5.68)	1241 (5.48)	1437 (5.84)	1625 (6.10)	1786 (6.33)	1842 (6.16)	1947 (5.94)
α-Glucosidase inhibitor (number (%))	4289 (24.2)	4211 (22.4)	4268 (21.4)	4307 (20.3)	4260 (18.8)	4119 (16.7)	4370 (16.4)	4424 (15.7)	4314 (14.4)	4200 (12.8)
Biguanide (number (%))	2717 (15.3)	3028 (16.1)	3533 (17.7)	4057 (19.1)	4827 (21.3)	5823 (23.7)	6896 (25.9)	7841 (27.8)	8588 (28.7)	9478 (28.9)
Dipeptidyl peptidase-4 inhibitor (number (%))	10216 (57.6)	12184 (64.8)	13872 (69.5)	15282 (71.9)	16838 (74.3)	18551 (75.4)	20433 (76.7)	21594 (76.5)	22469 (75.1)	23718 (72.3)
Glucagon-like peptide-1 receptor agonist (number (%))	27 (0.152)	51 (0.271)	83 (0.416)	171 (0.804)	264 (1.16)	384 (1.56)	529 (1.99)	753 (2.67)	1077 (3.60)	1366 (4.17)
Sodium-coupled glucose transporter 2 inhibitor (number (%))	0 (0)	82 (0.436)	337 (1.69)	741 (3.49)	1383 (6.10)	2222 (9.03)	3816 (14.3)	5267 (18.7)	7551 (25.2)	10513 (32.1)
Thiazolidines (number (%))	2147 (12.1)	2084 (11.1)	2118 (10.6)	2107 (9.91)	2135 (9.42)	2146 (8.72)	2170 (8.15)	2195 (7.78)	2108 (7.05)	2115 (6.45)
Hypertension (number (%))	13730 (77.4)	14501 (77.1)	15334 (76.8)	16217 (76.3)	17328 (76.5)	18763 (76.3)	20283 (76.2)	21530 (76.3)	22971 (76.8)	25409 (77.5)
Dyslipidemia (number (%))	9142 (51.5)	9988 (53.1)	10836 (54.3)	11645 (54.8)	12527 (55.3)	13885 (56.5)	15360 (57.7)	16662 (59.1)	18020 (60.2)	20146 (61.5)

**Table 4:** Characteristics of HCN subjects and the proportion of drug prescriptions and hypoglycemia occurrence by year HCN: having care needs group

Fiscal year	2013	2014	2015	2016	2017	2018	2019	2020	2021	2022
Total subjects (number)	4331	4604	4949	5261	5476	5347	5317	5373	5215	5217
Male (number (%))	1729 (39.9)	1839 (39.9)	2031 (41.0)	2189 (41.6)	2280 (41.6)	2188 (40.9)	2147 (40.4)	2202 (41.0)	2073 (39.8)	2016 (38.6)
Mean age (years)	84.3	84.6	84.8	84.9	84.9	85.0	85.1	85.2	85.7	86.2
Age 75-79 (number (%))	1085 (25.1)	1097 (23.8)	1144 (23.1)	1190 (22.6)	1236 (22.6)	1141 (21.3)	1201 (22.6)	1148 (21.4)	957 (18.4)	781 (15.0)
Age 80-84 (number (%))	1320 (30.5)	1396 (30.3)	1518 (30.7)	1568 (29.8)	1609 (29.4)	1549 (29.0)	1484 (27.9)	1556 (29.0)	1432 (27.5)	1383 (26.5)
Age 85-89 (number (%))	1222 (28.2)	1310 (28.5)	1388 (28.0)	1476 (28.1)	1529 (27.9)	1547 (28.9)	1501 (28.2)	1493 (27.8)	1628 (31.2)	1688 (32.4)
Age 90-94 (number (%))	558 (12.9)	642 (13.9)	687 (13.9)	815 (15.5)	856 (15.6)	870 (16.3)	869 (16.3)	904 (16.8)	885 (17.0)	1011 (19.4)
Age ≥ 95 (number (%))	146 (3.37)	159 (3.45)	212 (4.28)	212 (4.03)	246 (4.49)	240 (4.49)	262 (4.93)	272 (5.06)	313 (6.00)	354 (6.79)
Hypoglycemia (number (%))	54 (1.25)	69 (1.50)	57 (1.15)	59 (1.12)	57 (1.04)	52 (0.973)	50 (0.940)	55 (1.02)	41 (0.786)	43 (0.824)
Insulin (all) (number (%))	805 (18.6)	804 (17.5)	837 (16.9)	826 (15.7)	841 (15.4)	768 (14.4)	741 (13.9)	728 (13.5)	659 (12.6)	664 (12.7)
Insulin (intermediate-acting) (number (%))	400 (9.24)	369 (8.01)	350 (7.07)	288 (5.47)	244 (4.46)	185 (3.46)	169 (3.18)	123 (2.29)	104 (1.99)	98 (1.88)
Insulin (long-acting) (number (%))	320 (7.39)	353 (7.67)	412 (8.32)	481 (9.14)	519 (9.48)	524 (9.80)	521 (9.80)	540 (10.1)	479 (9.19)	509 (9.76)
Insulin (rapid-acting) (number (%))	528 (12.2)	499 (10.8)	498 (10.1)	456 (8.67)	492 (8.98)	437 (8.17)	413 (7.77)	377 (7.02)	358 (6.86)	345 (6.61)
Sulfonylurea (number (%))	1606 (37.1)	1528 (33.2)	1495 (30.2)	1421 (27.0)	1419 (25.9)	1190 (22.3)	1090 (20.5)	1021 (19.0)	930 (17.8)	849 (16.3)
Glinide (number (%))	168 (3.88)	190 (4.13)	210 (4.24)	228 (4.33)	235 (4.29)	258 (4.83)	290 (5.45)	300 (5.58)	287 (5.50)	284 (5.44)
α-Glucosidase inhibitor (number (%))	838 (19.3)	822 (17.9)	811 (16.4)	790 (15.0)	800 (14.6)	719 (13.4)	687 (12.9)	599 (11.1)	544 (10.4)	527 (10.1)
Biguanide (number (%))	373 (8.61)	396 (8.60)	485 (9.80)	607 (11.5)	687 (12.5)	736 (13.8)	860 (16.2)	965 (18.0)	985 (18.9)	997 (19.1)
Dipeptidyl peptidase-4 inhibitor (number (%))	2324 (53.7)	2927 (63.6)	3435 (69.4)	3880 (73.8)	4161 (76.0)	4098 (76.6)	4132 (77.7)	4143 (77.1)	3945 (75.6)	3855 (73.9)
Glucagon-like peptide-1 receptor agonist (number (%))	12 (0.277)	10 (0.217)	18 (0.364)	53 (1.01)	82 (1.50)	138 (2.58)	169 (3.18)	225 (4.19)	252 (4.83)	286 (5.48)
Sodium-coupled glucose transporter 2 inhibitor (number (%))	0 (0)	12 (0.261)	56 (1.13)	114 (2.17)	233 (4.25)	321 (6.00)	507 (9.54)	653 (12.2)	923 (17.7)	1273 (24.4)
Thiazolidines (number (%))	361 (8.34)	351 (7.62)	352 (7.11)	336 (6.39)	335 (6.12)	330 (6.17)	295 (5.55)	276 (5.14)	250 (4.79)	240 (4.60)
Hypertension (number (%))	3336 (77.0)	3522 (76.5)	3744 (75.7)	3963 (75.3)	4149 (75.8)	4014 (75.1)	4007 (75.4)	4010 (74.6)	4001 (76.7)	4038 (77.4)
Dyslipidemia (number (%))	1560 (36.0)	1637 (35.6)	1859 (37.6)	2061 (39.2)	2215 (40.4)	2193 (41.0)	2165 (40.7)	2203 (41.0)	2266 (43.5)	2340 (44.9)

**Table 5:** Age-adjusted proportion of drug prescriptions and hypoglycemia occurrence by year in NCN NCN: no care needs group

Fiscal year	2013	2014	2015	2016	2017	2018	2019	2020	2021	2022
Hypoglycemia (%)	0.467	0.401	0.434	0.406	0.231	0.220	0.189	0.200	0.196	0.250
Insulin (all) (%)	13.8	13.1	12.6	12.2	12.1	11.9	11.4	11.3	10.8	10.2
Insulin (intermediate-acting) (%)	7.74	6.68	5.69	4.56	3.73	3.18	2.72	2.51	2.06	1.74
Insulin (long-acting) (%)	5.01	5.41	5.98	6.75	7.35	7.84	7.86	7.89	8.00	7.71
Insulin (rapid-acting) (%)	9.64	8.85	8.17	7.95	8.01	7.73	7.34	7.28	6.60	6.12
Sulfonylurea (%)	41.5	38.2	35.7	32.5	30.4	26.6	25.0	23.2	21.1	19.0
Glinide (%)	4.81	4.56	5.04	5.51	5.30	5.60	5.90	6.18	6.03	5.85

**Table 6:** Age-adjusted proportion of drug prescriptions and hypoglycemia occurrence by year in HCN HCN: having care needs group

Fiscal year	2013	2014	2015	2016	2017	2018	2019	2020	2021	2022
Hypoglycemia (%)	1.17	1.36	1.18	1.04	1.11	1.01	0.995	1.15	0.671	0.770
Insulin (all) (%)	19.9	18.4	18.2	16.9	16.8	15.8	15.2	14.7	13.8	13.9
Insulin (intermediate-acting) (%)	9.85	8.34	7.48	5.82	4.87	3.73	3.43	2.47	2.15	1.92
Insulin (long-acting) (%)	7.90	8.15	9.04	9.88	10.4	10.8	10.7	10.9	10.0	10.8
Insulin (rapid-acting) (%)	13.3	11.6	11.0	9.68	10.2	9.45	8.91	7.92	7.60	7.33
Sulfonylurea (%)	36.0	32.6	29.5	26.4	25.3	21.8	20.0	18.7	17.7	16.4
Glinide (%)	4.07	4.27	4.55	4.47	4.36	5.20	6.03	6.05	5.91	5.95

## Discussion

Individualized blood glucose control targets should be set to prevent the risk of hypoglycemia when treating diabetes in older adults, considering age, ADL, presence of dementia, and diabetes medicines used. Hypoglycemia can lead to dementia, falls, fractures, cardiovascular disease, microvascular disease, and even death [17]. However, diabetes management in older adults who need care may not be optimal in many parts of the world. For example, some previous research showed that diabetes management in nursing home residents may not be appropriate [7-9]. Moreover, in Germany, there was no difference reported in glycemic management status between nursing homes and home care, with approximately 50% of patients having an HbA1c of less than 7%. In brief, it was implied that diabetes management in older adults who need care was suboptimal, regardless of residence [10]. Some studies have reported the glycemic management status of patients with diabetes requiring care. However, similar studies have not been conducted in Japan. No study has compared the incidence of hypoglycemia between patients with diabetes who do and do not require care. This study is novel in that it examined the prescription status of diabetes medicines and the incidence of hypoglycemia in older adults at each level of care need, using a database that links medical and care information. In particular, for older adults aged 75 years and above, the Nara KDB made it possible to survey almost all people in terms of medical and care data, thereby minimizing selection bias.

In Japan, in May 2017, for the first time, guidelines set targets for glycemic control in older adults, considering age, ADL, and the presence of dementia. According to this guideline, for patients using SUs, GLs, and insulin, a higher target for HbA1c control was set to avoid hypoglycemia, and a lower limit was set. Of the three drugs (insulin, SUs, and GLs) that pose a high risk of hypoglycemia when used alone, the proportion of prescriptions for insulin and SUs decreased monotonically from 2013 to 2022. The "Managing Older People With Type 2 Diabetes: Global Guidelines" was published by the International Diabetes Federation in 2013, preceding the Japanese guidelines. This may explain why the proportion of prescriptions for SUs and insulin decreased before 2017. GLs are classified as drugs with a high risk of hypoglycemia when used alone because they have a similar mechanism of blood glucose-lowering effect to SUs; however, they are relatively safe to use due to their shorter duration of action than SUs. Actually, it was shown that repaglinide (one of GLs) experienced fewer hypoglycemic events compared with glibenclamide (one of SUs)-treated patients, with similar metabolic control [18]. This may have led some patients to switch from SUs to GLs and can explain the increase in the proportion of GL prescriptions.

When analyzing the proportion of insulin prescriptions by duration of action in three categories (RIs, IIs, and LIs), the proportion of prescriptions for RIs and IIs decreased from 2013 to 2022. However, the proportion of prescriptions for LIs increased. Some older people with diabetes may have reduced endogenous insulin secretory capacity owing to age or other reasons [19], and treatment with LIs plus RIs or LIs plus diabetes drugs other than insulin is thus often selected. This can explain why the decreasing trend was not observed for LIs. One possible explanation for the slightly increasing trend in the proportion of prescriptions of LIs could be that patients with insulin-dependent conditions who were originally prescribed IIs are now being switched to LIs owing to a longer duration of action and a more stable effect. LIs are more beneficial than IIs in people with diabetes who are at a high risk of hypoglycemia [9]. Another possible explanation could be that many patients develop contraindications to other antidiabetic medications (such as renal insufficiency), which makes LIs a more favorable option with age. LIs are preferred over other types of insulin due to their prolonged duration of action and ease of administration, as they typically require only a once-daily injection, which is more manageable for caregivers. Additionally, given that glycemic targets are often less stringent in older adults, adequate glycemic control can frequently be achieved with LIs alone, without the need for prandial insulin. The reason why the proportion of prescriptions for GLP1RAs and SGLT2Is (GLP1RAs: 0.177% to 4.35%, SGLT2Is: 0% to 31%) was increasing rapidly from 2013 to 2022 may be related to the growing body of evidence supporting their additional benefits, including a reduction in cardiovascular and renal complications, as well as a more favorable safety profile compared to older drugs [20-22]. The percentage of SGLT2Is prescribed in 2013 was 0%, because the first SGLT2Is were launched in Japan in 2014. The proportion of prescriptions for BGs and DPP4Is also showed an increasing trend. These drugs are less likely to cause hypoglycemia when used alone, so it was thought that they were preferred for prescription to older adults who were at high risk of hypoglycemia.

The major difference observed between NCN and HCN was in terms of the incidence of hypoglycemia. The incidence of hypoglycemia decreased between 2013 and 2022 in both groups; however, in both years, the incidence of hypoglycemia was higher in HCN than in NCN. The main reason for the difference in the incidence of hypoglycemia between HCN and NCN was as follows: HCN was more prone to hypoglycemia than NCN. For example, older adults in HCN are likely to be frail. Frailty associated with physical and cognitive decline warrants the need for care and is correlated with worsening nutritional status [23,24]. Furthermore, it is thought that older adults in HCN tend to be prescribed insulin more often than other diabetes medications. One previous study reported that older adults in nursing homes needed higher-level care than those who received home care [25]. Another previous study reported that older adults in nursing homes were prescribed insulin more often than those who received home care [10]. The results of this study showed that HCN had a lower proportion of prescriptions for SUs and a higher proportion of prescriptions for insulin than NCN. This can explain the higher incidence of hypoglycemia in patients with HCN. It has previously been reported that insulin most likely causes hypoglycemia among all diabetic drugs [9]. The results of this study also supported that.

After 2017, the rate of decrease in the proportion of prescriptions for IIs, RIs, and SUs was slower than that before 2017, as was the rate of decrease in the incidence of hypoglycemia in NCN. Guidelines for the treatment of diabetes in older adults were published in Japan in 2017, preceded by international guidelines in 2013. Therefore, in the NCN, several occurrences of hypoglycemia due to inappropriate diabetes treatment had been avoided by 2017, after which the decline in the incidence of hypoglycemia plateaued. In contrast, in HCN, despite a slowdown in the decrease in the proportion of prescriptions for SUs and insulin, the rate of decrease in the incidence of hypoglycemia was higher after 2017 than before 2017. This may be because blood glucose control targets were more relaxed to avoid the risk of hypoglycemia after the publication of the 2017 guidelines in Japan, leading to a reduction in the dosage of SUs and insulin, while still being prescribed.

In Japan, as of 2020, the prevalence of hypertension among older adults aged 75 and over is estimated to be 34.5%, and the prevalence of dyslipidemia is estimated to be 6.38% [26]. Although the prevalence of them was higher in the subjects of this study, it has been reported that people with diabetes have a higher prevalence of them than the general population [27,28]. Therefore, this result was consistent.

The strengths of this study include the following: this was the first study that clarified the prescription of diabetes medication and the incidence of hypoglycemia by care level through linking medical insurance and LTCI data, and the possibility of selection bias was low because this study included almost all people aged 75 and over in Nara Prefecture.

The limitations of this study include the following: blood test data, such as HbA1c and blood glucose levels, were not included; prescription status was at the end of each year, so drug changes within the same year were not considered; medication dosage was unknown; information on the use of nursing homes was not included; individual comorbidities were not evaluated; comparability between NCN and HCN might be low because there was a possibility that confounding factors had not been controlled; and people with a condition corresponding to HCN but who did not apply for LTCI may have been included under NCN.

## Conclusions

In Japan, diabetes treatment for older adults has been optimized as an international trend since 2017, regardless of the care level. The incidence of hypoglycemia is on a decreasing trend overall. Moreover, this trend has been maintained since 2017. Individuals needing higher levels of care experienced more hypoglycemia than those requiring lower levels of care, a difference that has not been bridged to date. This observation may be due to greater frailty and conditions in which insulin is more likely to be prescribed among people receiving higher levels of care. Our findings suggest the need for further individualized treatment strategies and close monitoring.

## Appendices

List of ICD-10 codes

*Diabetes (Excluding Type 1 Diabetes* *)*

E10, E11, E12, E13, E14, E15, E110, E111, E112, E113, E114, E115, E116, E117, E119, E130, E131, E132, E133, E134, E135, E136, E137, E139, E140, E141, E142, E143, E144, E145, E146, E149, E741, E748, E831, E888, E891, N189, O241, O244, O249, O998, P700, P701, P702, P708, R81, R730, R739

Type 1 Diabetes

E10, E100, E101, E102, E103, E104, E105, E106, E107, E109, O240

Hypoglycemia

E15, E100, E110, E140, E160, E161, E162, E711, P703, P704

Hypertension

D350, F453, I10, I150, I151, I152, I159, O13, O16, O100, O104, O140, O141, O149, P292

Dyslipidemia

E780, E781, E782, E783, E784, E785, E786, E788, E789

List of Japanese disease codes

Diabetes (Excluding Type 1 Diabetes)

2500001, 2500013, 2500015, 2500024, 2500027, 2500031, 2500037, 2500041, 2501002, 2501003, 2501005, 2502004, 2502006, 2503005, 2503007, 2504004, 2504005, 2504006, 2504010, 2504012, 2504013, 2505011, 2505018, 2505021, 2506006, 2506011, 2507025, 2507028, 2507029, 2509003, 2509004, 2713009, 2714002, 6489003, 7751001, 7751002, 7915002, 7915003, 8830039, 8830040, 8830041, 8830042, 8830043, 8830044, 8830045, 8830405, 8830756, 8831132, 8831399, 8831401, 8833419, 8833420, 8834843, 8835244, 8835464, 8835685, 8835871, 8835941, 8836104, 8836563, 8838062, 8838063, 8838064, 8838065, 8838066, 8838067, 8838068, 8838069, 8838070, 8838071, 8838072, 8838073, 8838074, 8838075, 8838076, 8838077, 8838078, 8838079, 8838080, 8838081, 8838619, 8838621, 8838633, 8839324, 8840104, 8840710, 8841021, 8841689, 8841690, 8841691, 8841692, 8841693, 8841694, 8841695, 8841696, 8841697, 8841698, 8843106, 8843120, 8843121, 8843122, 8843123, 8843124, 8843125, 8843126, 8843127, 8843128, 8843375, 8843376, 8843377, 8843378, 8843379, 8843380, 8843381, 8843382, 8843383, 8843388, 8843389, 8843390, 8843391, 8843392, 8843393, 8843394, 8843395, 8843396, 8843439, 8843448, 8843449, 8843450, 8843451, 8843452, 8843453, 8843454, 8843455, 8843456, 8843619, 8843620, 8843621, 8843622, 8843623, 8843624, 8843625, 8843626, 8843627, 8843990, 8843991, 8843992, 8843993, 8843994, 8843995, 8843996, 8843997, 8844089, 8844233, 8844347, 8844537, 8844628, 8844629, 8844652, 8844653, 8845072, 8845073, 8845074, 8845075, 8845076, 8845077, 8845078, 8845079, 8845080, 8845081, 8845082, 8845083, 8845084, 8845085, 8845086, 8845087, 8845088, 8845089, 8845090, 8845091, 8845092, 8845093, 8845094, 8845095, 8845096, 8845097, 8845098, 8845099, 8845100, 8845128, 8845198, 8848108, 8848632, 8848633, 8848634, 8848768, 8849058, 8849181, 8849469, 8849470, 8849471, 8849472, 8849473, 8849474, 8849475, 8849476, 8849477, 8849478, 8849558, 8849585, 8849586, 8849587, 8849588, 8849589, 8849590, 8849591, 8849592, 8849593, 8849594, 8849874, 8849976, 8850065, 8851031

Type 1 Diabetes

2500014, 8830028, 8830029, 8830030, 8830031, 8830032, 8830033, 8841679, 8841680, 8841681, 8841682, 8841683, 8841684, 8841685, 8841686, 8841687, 8841688, 8843105, 8843982, 8843983, 8843984, 8843985, 8843986, 8843987, 8843988, 8843989, 8844022, 8844023, 8844024, 8844025, 8844026, 8844027, 8844028, 8844029, 8844030, 8844031, 8844045, 8844346, 8844536, 8844626, 8844627, 8845043, 8845044, 8845045, 8845046, 8845047, 8845048, 8845049, 8845050, 8845051, 8845052, 8845053, 8845054, 8845055, 8845056, 8845057, 8845058, 8845059, 8845060, 8845061, 8845062, 8845063, 8845064, 8845065, 8845066, 8845067, 8845068, 8845069, 8845070, 8845071, 8845842, 8849056, 8849557

Hypoglycemia

2510003, 2512004, 2512009, 7756001, 7756003, 8830430, 8830649, 8834798, 8837871, 8837872, 8838076, 8839324, 8840698, 8841208, 8844963, 8845065, 8845094

Hypertension

4019016, 4019017, 6429003, 8830212, 8832479, 8833421, 8834071, 8834893, 8835022, 8835586, 8835605, 8835614, 8838336, 8838398, 8838596, 8839689, 8840107, 8842089, 8842094, 8842178, 8842488, 8842500, 8842709, 8842726, 8842765, 8842791, 8842804, 8842827, 8844144, 8845461, 8845462, 8848337, 8849300, 8849491

Dyslipidemia

2720001, 2720004, 2721002, 2723001, 2724007, 2724012, 2724023, 2724031, 2724036, 2724037, 2729002, 2729003, 3335006, 8830371, 8831264, 8831269, 8831270, 8831271, 8831272, 8831273, 8831274, 8831275, 8831286, 8833120, 8833435, 8833663, 8833722, 8833881, 8837852, 8837884, 8840108, 8840506, 8840985, 8842204, 8844446, 8845523, 8845524, 8848266, 8848306, 8849518, 8849528, 8849534, 8849543, 8849690, 8849849, 8850975, 8851069, 8851070, 8851146, 8851147, 8851149

List of Japanese drug codes

Drug for Diabetes

610406257, 610406276, 610406357, 610406390, 610406391, 610407055, 610409349, 610409350, 610411045, 610412056, 610432026, 610432027, 610432032, 610432033, 610432040, 610432041, 610433079, 610441042, 610441043, 610443002, 610443003, 610444147, 610453088, 610461124, 610463145, 613960002, 613960003, 613960004, 613960006, 613960008, 613960009, 613960011, 613960012, 613960014, 613960015, 613960016, 613960017, 613960018, 613960019, 613960020, 613960024, 613960025, 613960026, 613960027, 613960028, 613960032, 613960036, 613960037, 613960038, 613960039, 613960040, 613960041, 613960047, 613960048, 613960050, 613960051, 613960053, 613960054, 613960057, 613960058, 613960060, 613960061, 613960062, 613960063, 613960064, 613960067, 613960068, 613960069, 613960070, 613960071, 613960072, 613960073, 613960074, 613960075, 613960076, 613960077, 613960078, 613960079, 613960080, 613960081, 613960082, 620000048, 620000202, 620000203, 620000204, 620000205, 620000265, 620000266, 620000267, 620000268, 620000269, 620000270, 620000271, 620000442, 620000443, 620000447, 620000448, 620000732, 620001907, 620001908, 620002029, 620002030, 620002031, 620002032, 620002120, 620002121, 620002439, 620002440, 620002441, 620002442, 620002443, 620002444, 620002445, 620002663, 620002665, 620002666, 620002667, 620002668, 620002710, 620002717, 620002730, 620002731, 620002810, 620002811, 620002812, 620002813, 620002815, 620002816, 620002824, 620002825, 620002826, 620002827, 620002828, 620002829, 620002835, 620002836, 620002837, 620002838, 620002839, 620002840, 620002841, 620002842, 620002843, 620002844, 620002845, 620002846, 620002847, 620002848, 620002859, 620002862, 620003127, 620003128, 620003129, 620003143, 620003144, 620003145, 620003159, 620003160, 620003277, 620003452, 620003568, 620003604, 620003661, 620003947, 620003948, 620004045, 620004046, 620004069, 620004070, 620004071, 620004072, 620004073, 620004074, 620004289, 620004480, 620004482, 620004502, 620004580, 620004781, 620004842, 620005359, 620005360, 620005557, 620005558, 620005559, 620005560, 620005561, 620005562, 620005563, 620005564, 620005565, 620005566, 620005570, 620005885, 620005900, 620005901, 620005979, 620006030, 620006590, 620006682, 620006683, 620006872, 620006890, 620006891, 620007459, 620007460, 620007461, 620007462, 620007536, 620008071, 620008072, 620008073, 620008074, 620008075, 620008076, 620008726, 620008727, 620008728, 620008729, 620008893, 620008894, 620008895, 620008896, 620008897, 620008898, 620008899, 620008907, 620008908, 620008909, 620008910, 620008911, 620008912, 620008913, 620008914, 620008915, 620008916, 620008932, 620008933, 620008934, 620008935, 620008936, 620008942, 620008943, 620008944, 620008945, 620008952, 620008953, 620009133, 620009209, 620009286, 620009287, 620009288, 620009289, 620009290, 620009291, 620009292, 620009293, 620009294, 620009295, 620009296, 620009297, 620871407, 620871601, 620871907, 620872001, 620872002, 620872003, 620872004, 620872009, 620872016, 620872304, 620873202, 620873301, 620873402, 620873702, 620873901, 621665301, 621665401, 621665502, 621673401, 621673501, 621673601, 621676001, 621678801, 621678901, 621679001, 621682201, 621682301, 621682402, 621682502, 621682603, 621683201, 621683401, 621683501, 621689001, 621689101, 621689303, 621689403, 621690203, 621690303, 621690402, 621690502, 621690901, 621691001, 621691101, 621691201, 621691501, 621691601, 621748301, 621784902, 621785002, 621896402, 621896502, 621911101, 621911201, 621911301, 621926901, 621927001, 621937101, 621937201, 621942101, 621942102, 621942201, 621942202, 621943301, 621943401, 621950901, 621951001, 621951101, 621953301, 621953401, 621958701, 621958801, 621970601, 621970701, 621970801, 621973201, 621973301, 621974701, 621974801, 621980701, 621982701, 621986001, 621986101, 621986201, 621986301, 621986401, 621990901, 621991001, 621997001, 621997101, 621998701, 621998801, 621998901, 621999001, 621999301, 621999401, 621999701, 621999801, 622000601, 622000701, 622001701, 622001801, 622004701, 622004702, 622004801, 622004802, 622005501, 622005601, 622005801, 622005802, 622008501, 622008502, 622008601, 622008602, 622008701, 622008801, 622009801, 622009802, 622009901, 622010001, 622011401, 622011501, 622011601, 622011701, 622013401, 622013501, 622013601, 622016001, 622016101, 622017301, 622017401, 622017501, 622017901, 622018001, 622018801, 622018802, 622020901, 622020903, 622021001, 622021003, 622021801, 622021901, 622022001, 622022101, 622023501, 622023601, 622025201, 622025301, 622025801, 622025901, 622026501, 622026601, 622029901, 622030001, 622031401, 622031501, 622033001, 622033101, 622033201, 622033701, 622033801, 622035701, 622035801, 622036001, 622036002, 622037901, 622038001, 622038301, 622038401, 622038801, 622038901, 622039901, 622040901, 622041001, 622041202, 622041302, 622041402, 622041502, 622042901, 622043001, 622045201, 622045301, 622045401, 622045501, 622046801, 622046901, 622047701, 622047801, 622048401, 622048501, 622049901, 622050001, 622053101, 622053201, 622053601, 622053801, 622055801, 622055901, 622056001, 622056101, 622058801, 622058901, 622059001, 622059002, 622059101, 622059102, 622059201, 622059301, 622061001, 622061401, 622061501, 622061601, 622061701, 622062301, 622062302, 622062401, 622062402, 622063001, 622063101, 622063201, 622063301, 622065101, 622065201, 622065301, 622065401, 622066201, 622066301, 622070801, 622071701, 622071801, 622071901, 622072001, 622075601, 622078301, 622078401, 622079101, 622079201, 622081801, 622081901, 622086001, 622086101, 622088001, 622088301, 622088401, 622090001, 622090101, 622093501, 622103201, 622114401, 622114501, 622114601, 622114701, 622114801, 622118501, 622119301, 622119401, 622122201, 622122301, 622127301, 622127401, 622127501, 622128101, 622137701, 622141101, 622141301, 622141302, 622143401, 622143402, 622144001, 622144601, 622144701, 622147301, 622147401, 622147501, 622147601, 622155701, 622155801, 622155901, 622156001, 622156901, 622157001, 622159301, 622159401, 622159501, 622163301, 622163401, 622164301, 622164401, 622166801, 622166901, 622167201, 622167301, 622169101, 622169102, 622169301, 622171301, 622172101, 622172201, 622175401, 622175501, 622175601, 622175701, 622176301, 622177501, 622178601, 622178701, 622182401, 622182501, 622182601, 622186201, 622187301, 622190001, 622190002, 622190801, 622193301, 622194901, 622196601, 622196701, 622198001, 622198901, 622199001, 622201701, 622202201, 622202801, 622205101, 622205501, 622208901, 622211501, 622217701, 622219701, 622221001, 622222001, 622229001, 622230001, 622230101, 622242001, 622242501, 622245601, 622245701, 622246801, 622252501, 622252701, 622254701, 622267001, 622271101, 622271201, 622271301, 622277501, 622288401, 622302201, 622302301, 622306601, 622306701, 622311100, 622313200, 622313300, 622320800, 622320900, 622335701, 622335801, 622336801, 622338501, 622338601, 622338701, 622340101, 622341901, 622342001, 622360601, 622401201, 622401301, 622406001, 622410901, 622411001, 622412701, 622415401, 622415501, 622417101, 622417201, 622421101, 622421201, 622421901, 622422001, 622424401, 622424501, 622426601, 622426701, 622427201, 622427301, 622432501, 622432601, 622432701, 622436301, 622438401, 622438501, 622440701, 622442201, 622448601, 622448901, 622449001, 622450301, 622450401, 622450901, 622451001, 622462401, 622462501, 622466601, 622484801, 622515201, 622515301, 622517101, 622518101, 622518102, 622518201, 622518202, 622520901, 622521001, 622523301, 622523401, 622525301, 622525401, 622544301, 622544401, 622544501, 622560201, 622560301, 622560401, 622573601, 622625702, 622628601, 622628701, 622628801, 622631401, 622636501, 622636601, 622642701, 622654401, 622654501, 622655001, 622655101, 622660601, 622662501, 622662601, 622662701, 622662801, 622664101, 622664201, 622664301, 622671001, 622691700, 622699501, 622740200, 622740300, 622740400, 622740500, 622740600, 622740700, 622740800, 622740900, 622741000, 622741100, 622741200, 622741300, 622741400, 622784601, 622784701, 622795901, 622796001, 622813401, 622813501, 622822401, 622822501, 622847100, 622861201, 622861301, 622869901, 629900701, 629905601, 629905701, 629905801, 629906701, 629906801, 629906901, 629907001, 629907301, 629907401, 629907501, 629907601, 629907701, 629907801, 629907901, 629911601, 629911701, 629911801, 629914101, 629914201, 640406238, 640406239, 640406240, 640407062, 640407063, 640407220, 640407221, 640407222, 640412080, 640412081, 640412082, 640412083, 640412084, 640412085, 640422059, 640422067, 640422068, 640422069, 640422074, 640451027, 640451028, 640451029, 640451038, 640451039, 640451040, 640451041, 640453021, 640453022, 640453023, 640453031, 642490058, 642490059, 642490060, 642490061, 642490063, 642490064, 642490065, 642490066, 642490068, 642490069, 642490070, 642490071, 642490092, 642490094, 642490095, 642490097, 642490099, 642490100, 642490101, 642490102, 642490103, 642490104, 642490106, 642490107, 642490108, 642490109, 642490111, 642490112, 642490115, 642490120, 642490121, 642490122, 642490123, 642490124, 642490125, 642490126, 642490127, 642490128, 642490129, 642490130, 642490131, 642490132, 642490133, 642490134, 642490135, 642490136, 642490137, 642490138

Insulin

620000202, 620000203, 620000204, 620000205, 620000265, 620000266, 620000267, 620000268, 620000269, 620000270, 620000271, 620000442, 620000443, 620000447, 620000448, 620002439, 620002440, 620002441, 620002442, 620002443, 620002444, 620002445, 620004781, 620005900, 620005901, 620007459, 620007460, 620007461, 620007462, 620007536, 620008893, 620008894, 620008895, 620008896, 620008897, 620008898, 620008899, 620008907, 620008908, 620008909, 620008910, 620008911, 620008912, 620008913, 620008914, 620008915, 620008916, 620008932, 620008933, 620008934, 620008935, 620008936, 620008942, 620008943, 620008944, 620008945, 620008952, 620008953, 621911101, 621911201, 621911301, 621926901, 621927001, 621973201, 621973301, 622114401, 622114501, 622114601, 622198901, 622199001, 622252701, 622410901, 622411001, 622440701, 622450901, 622451001, 622484801, 622642701, 629900701, 629905601, 629905701, 629905801, 629906701, 629906801, 629906901, 629907001, 629907301, 629907401, 629907501, 629907601, 629914101, 629914201, 640406238, 640406239, 640406240, 640407062, 640407063, 640407220, 640407221, 640407222, 640412080, 640412081, 640412082, 640412083, 640412084, 640412085, 640422059, 640422067, 640422068, 640422069, 640422074, 640451027, 640451028, 640451029, 640451038, 640451039, 640451040, 640451041, 640453021, 640453022, 640453023, 640453031, 642490058, 642490059, 642490060, 642490061, 642490063, 642490064, 642490065, 642490066, 642490068, 642490069, 642490070, 642490071, 642490092, 642490094, 642490095, 642490097, 642490099, 642490100, 642490101, 642490102, 642490103, 642490104, 642490106, 642490107, 642490108, 642490109, 642490111, 642490112, 642490115, 642490120, 642490121, 642490122, 642490123, 642490124, 642490125, 642490126, 642490127, 642490128, 642490129, 642490130, 642490131, 642490132, 642490133, 642490134, 642490135, 642490136, 642490137, 642490138

50% or 70% Glucose

620002599, 620006649, 640412069, 640412070, 640412071, 640460006, 643230048, 643230049, 643230050, 643230052, 643230252

Drug for Hypertension

610406002, 610406009, 610406012, 610406015, 610406028, 610406029, 610406030, 610406053, 610406062, 610406063, 610406101, 610406119, 610406131, 610406132, 610406157, 610406158, 610406167, 610406168, 610406191, 610406214, 610406216, 610406217, 610406218, 610406219, 610406233, 610406274, 610406284, 610406310, 610407003, 610407004, 610407005, 610407006, 610407010, 610407011, 610407019, 610407020, 610407021, 610407046, 610407069, 610407090, 610407115, 610407142, 610407143, 610407144, 610407147, 610407148, 610407149, 610407150, 610407151, 610407152, 610407348, 610407349, 610407466, 610407467, 610408605, 610409327, 610409328, 610409329, 610409331, 610409332, 610409333, 610412007, 610412012, 610412013, 610412014, 610412032, 610412035, 610412036, 610412037, 610412038, 610412039, 610412040, 610412041, 610412044, 610412045, 610412046, 610412048, 610412054, 610412062, 610412065, 610412066, 610412067, 610412072, 610412073, 610412080, 610412081, 610412110, 610412111, 610412129, 610412130, 610412142, 610412143, 610412145, 610412176, 610412177, 610412184, 610421024, 610421318, 610421319, 610421320, 610421321, 610421322, 610421329, 610421330, 610421346, 610422032, 610422033, 610422046, 610422047, 610422063, 610422064, 610422102, 610422103, 610422110, 610422111, 610422121, 610422126, 610422142, 610422163, 610422164, 610422193, 610422194, 610422202, 610422203, 610422208, 610422209, 610422210, 610422211, 610422212, 610422213, 610422214, 610422215, 610422225, 610422226, 610422230, 610422231, 610422237, 610422238, 610431025, 610431115, 610432011, 610432012, 610432013, 610432014, 610433002, 610433007, 610433011, 610433016, 610433017, 610433018, 610433019, 610433028, 610433038, 610433039, 610433040, 610433041, 610433042, 610433064, 610433065, 610433066, 610433067, 610433068, 610433069, 610433089, 610433104, 610433113, 610433114, 610433133, 610433134, 610441014, 610441015, 610441016, 610443042, 610443043, 610443044,610444001, 610444002, 610444017, 610444019, 610444020, 610444021, 610444022, 610444023, 610444028, 610444029, 610444030, 610444031, 610444032, 610444033, 610444034, 610444035, 610444040, 610444049, 610444050, 610444062, 610444064, 610444065, 610444066, 610444067, 610444071, 610444072, 610444073, 610444074, 610444075, 610444076, 610444079, 610444080, 610444084, 610444085, 610444101, 610444119, 610444120, 610444124, 610444148, 610444153, 610444154, 610444158, 610444159, 610444160, 610444161, 610444162, 610444163, 610444164, 610444165, 610444166, 610444167, 610444169, 610444170, 610444172, 610444178, 610453013, 610453017, 610453018, 610453025, 610453036, 610453037, 610453060, 610453083, 610453089, 610453090, 610453097, 610453098, 610453109, 610453121, 610453122, 610453123, 610453124, 610453125, 610453126, 610453127, 610453129, 610453147, 610453148, 610453149, 610454035, 610454080, 610454081, 610460014, 610460017, 610461002, 610461003, 610461089, 610461090, 610461106, 610461108, 610461153, 610461156, 610461196, 610461204, 610461205, 610462038, 610462039, 610462040, 610462043, 610462044, 610463009, 610463010, 610463013, 610463014, 610463016, 610463027, 610463028, 610463039, 610463078, 610463100, 610463125, 610463126, 610463137, 610463138, 610463139, 610463140, 610463141, 610463142, 610463171, 610463187, 610463188, 610463211, 610463212, 610470001, 610470002, 612120001, 612120002, 612120004, 612120015, 612120016, 612120017, 612120020, 612120021, 612120022, 612120029, 612120030, 612120031, 612120033, 612120039, 612120054, 612120059, 612120060, 612120073, 612120080, 612120086, 612120092, 612120093, 612120094, 612120096, 612120106, 612120118, 612120119, 612120124, 612120128, 612120129, 612120134, 612120138, 612120141, 612120148, 612120165, 612120168, 612120171, 612120187, 612120224, 612120225, 612120226, 612120227, 612120238, 612120240, 612120247, 612120248, 612120249, 612120250, 612120251, 612120252, 612120256, 612120257, 612120258, 612120259, 612120266, 612120267, 612120309, 612120310, 612120312, 612120313, 612120317, 612120318, 612120319, 612120320, 612120321, 612120324, 612120330, 612120331, 612120334, 612120335, 612120336, 612120337, 612120338, 612120339, 612120341, 612120346, 612120347, 612120348, 612120349, 612120351, 612120356, 612120357, 612130025, 612130031, 612130037, 612130038, 612130039, 612130043, 612130044, 612130045, 612130046, 612130051, 612130052, 612130054, 612130055, 612130069, 612130070, 612130071, 612130074, 612130075, 612130080, 612130086, 612130091, 612130102, 612130104, 612130105, 612130110, 612130111, 612130112, 612130121, 612130125, 612130126, 612130131, 612130145, 612130156, 612130165, 612130168, 612130169, 612130176, 612130182, 612130188, 612130193, 612130198, 612130200, 612130202, 612130207, 612130211, 612130212, 612130217, 612130219, 612130220, 612130221, 612130223, 612130231, 612130235, 612130239, 612130240, 612130242, 612130249, 612130260, 612130261, 612130266, 612130267, 612130268, 612130270, 612130271, 612130272, 612130273, 612130274, 612130275, 612130276, 612130277, 612130294, 612130295, 612130327, 612130333, 612130335, 612130338, 612130339, 612130342, 612130344, 612130348, 612140033, 612140040, 612140070, 612140071, 612140073, 612140074, 612140075, 612140128, 612140129, 612140158, 612140159, 612140164, 612140204, 612140205, 612140206, 612140224, 612140233, 612140234, 612140235, 612140236, 612140244, 612140245, 612140246, 612140315, 612140316, 612140317, 612140327, 612140328, 612140423, 612140434, 612140435, 612140436, 612140437, 612140440, 612140443, 612140444, 612140445, 612140450, 612140451, 612140471, 612140472, 612140473, 612140474, 612140478, 612140479, 612140480, 612140481, 612140482, 612140483, 612140484, 612140485, 612140486, 612140487, 612140489, 612140490, 612140492, 612140493, 612140494, 612140495, 612140496, 612140497, 612140498, 612140499, 612140500, 612140501, 612140502, 612140503, 612140504, 612140516, 612140517, 612140518, 612140519, 612140520, 612140521, 612140522, 612140523, 612140524, 612140526, 612140527, 612140528, 612140529, 612140530, 612140531, 612140533, 612140534, 612140535, 612140536, 612140537, 612140538, 612140540, 612140541, 612140542, 612140543, 612140544, 612140545, 612140547, 612140548, 612140551, 612140552, 612140554, 612140555, 612140556, 612140557, 612140558, 612140559, 612140560, 612140561, 612140564, 612140565, 612140574, 612140576, 612140577, 612140579, 612140580, 612140590, 612140595, 612140601, 612140607, 612140614, 612140617, 612140619, 612140620, 612140623, 612140626, 612140630, 612140632, 612140633, 612140634, 612140637, 612140638, 612140644, 612140645, 612140647, 612140648, 612140649, 612140650, 612140651, 612140652, 612140654, 612140655, 612140656, 612140657, 612140658, 612140659, 612140660, 612140661, 612140662, 612140665, 612140666, 612140667, 612140670, 612140671, 612140674, 612140677, 612140678, 612140684, 612140685, 612140686, 612140693, 612140694, 612140695, 612140696, 612140697, 612140698, 612140699, 612140700, 612140701, 612140702, 612140703, 612140704, 612140705, 612140706, 612140707, 612140708, 612140711, 612140712, 612140713, 612140714, 612140715, 612140716, 612140718, 612140719, 612140720, 612140721, 612140722, 612170012, 612170014, 612170015, 612170039, 612170063, 612170072, 612170095, 612170209, 612170210, 612170212, 612170234, 612170268, 612170301, 612170339, 612170340, 612170341, 612170371, 612170381, 612170416, 612170424, 612170441, 612170449, 612170451, 612170452, 612170453, 612170457, 612170458, 612170469, 612170471, 612170472, 612170473, 612170474, 612170483, 612170484, 612170485, 612170539, 612170540, 612170543, 612170547, 612170549, 612170561, 612170594, 612170598, 612170610, 612170627, 612170631, 612170632, 612170633, 612170637, 612170647, 612170660, 612170661, 612170662, 612170663, 612170664, 612170665, 612170668, 612170669, 612170670, 612170671, 612170672, 612170673, 612170676, 612170677, 612170678, 612170679, 612170680, 612170681, 612170682, 612170683, 612170692, 612170693, 612170698, 612170699, 612170703, 612170704, 612170705, 612170706, 612170707, 612170708, 612170709, 612170710, 612190064, 612190065, 612190066, 612190103, 612190221, 612190222, 612190224, 612190225, 612190226, 612190227, 612190229, 612190230, 612190231, 612190232, 612190233, 612190234, 612190235, 612190236, 612190241, 612190242, 612190245, 612190247, 612190248, 612190249, 612190250, 620000005, 620000006, 620000040, 620000041, 620000068, 620000069, 620000075, 620000076, 620000077, 620000080, 620000081, 620000082, 620000126, 620000127, 620000131, 620000132, 620000133, 620000167, 620000168, 620000530, 620000531, 620000532, 620000533, 620001874, 620001875, 620001876, 620001877, 620001878, 620001881, 620001905, 620001906, 620001954, 620001955, 620001964, 620001990, 620001991, 620001999, 620002000, 620002005, 620002006, 620002007, 620002008, 620002009, 620002010, 620002011, 620002012, 620002013, 620002014, 620002015, 620002016, 620002017, 620002018, 620002019, 620002020, 620002021, 620002024, 620002025, 620002034, 620002035, 620002044, 620002045, 620002048, 620002053, 620002059, 620002060, 620002066, 620002067, 620002069, 620002070, 620002071, 620002072, 620002078, 620002079, 620002080, 620002082, 620002083, 620002086, 620002087, 620002095, 620002096, 620002106, 620002107, 620002132, 620002133, 620002134, 620002135, 620002136, 620002137, 620002138, 620002143, 620002144, 620002145, 620002146, 620002157, 620002158, 620002422, 620002429, 620002430, 620002468, 620002514, 620002543, 620002630, 620002700, 620002701, 620002706, 620002707, 620002708, 620002709, 620002718, 620002719, 620002720, 620002721, 620002723, 620002724, 620002732, 620002745, 620002746, 620002757, 620002758, 620002759, 620002760, 620002762, 620002819, 620002820, 620002821, 620002822, 620002855, 620002856, 620003082, 620003083, 620003132, 620003133, 620003178, 620003182, 620003262, 620003263, 620003439, 620003440, 620003441, 620003442, 620003510, 620003512, 620003525, 620003529, 620003560, 620003565, 620003580, 620003581, 620003587, 620003591, 620003612, 620003617, 620003618, 620003619, 620003620, 620003886, 620003887, 620003898, 620003899, 620003900, 620003901, 620003902, 620003903, 620003904, 620003905, 620003906, 620003907, 620003908, 620003909, 620003910, 620003915, 620003951, 620003952, 620003977, 620003978, 620004011, 620004012, 620004013, 620004014, 620004015, 620004016, 620004041, 620004042, 620004043, 620004047, 620004048, 620004049, 620004050, 620004051, 620004052, 620004053, 620004054, 620004055, 620004056, 620004057, 620004058, 620004059, 620004060, 620004061, 620004062, 620004063, 620004064, 620004065, 620004066, 620004283, 620004284, 620004286, 620004290, 620004389, 620004390, 620004414, 620004437, 620004443, 620004453, 620004467, 620004468, 620004470, 620004477, 620004478, 620004490, 620004527, 620004538, 620004539, 620004558, 620004559, 620004586, 620004593, 620004601, 620004615, 620004617, 620004844, 620004907, 620004914, 620004915, 620004919, 620004929, 620004938, 620004957, 620004966, 620004967, 620004986, 620005002, 620005003, 620005004, 620005005, 620005006, 620005019, 620005031, 620005049, 620005050, 620005074, 620005075, 620005076, 620005077, 620005097, 620005102, 620005119, 620005366, 620005429, 620005430, 620005523, 620005524, 620005550, 620005551, 620005552, 620005553, 620005554, 620005555, 620005556, 620005824, 620005825, 620005826, 620005854, 620005904, 620005905, 620005927, 620005935, 620005936, 620005953, 620005972, 620006003, 620006004, 620006050, 620006051, 620006056, 620006106, 620006110, 620006127, 620006128, 620006149, 620006155, 620006158, 620006556, 620006563, 620006564, 620006580, 620006585, 620006586, 620006597, 620006602, 620006664, 620006665, 620006745, 620006746, 620006747, 620006793, 620006794, 620006796, 620006797, 620006819, 620006820, 620006844, 620006857, 620006879, 620006932, 620007003, 620007010, 620007014, 620007015, 620007016, 620007070, 620007103, 620007160, 620007817, 620007818, 620007819, 620007820, 620007821, 620007822, 620007823, 620007824, 620007825, 620007826, 620007827, 620007828, 620007829, 620007830, 620007831, 620007832, 620007833, 620007834, 620007835, 620007836, 620007837, 620007838, 620007839, 620007840, 620007841, 620007842, 620007843, 620007844, 620007845, 620007846, 620007847, 620007848, 620007849, 620007850, 620007851, 620007852, 620007853, 620007854, 620007855, 620007856, 620007857, 620007858, 620007859, 620007860, 620007861, 620007862, 620007863, 620007864, 620007865, 620007866, 620007867, 620007868, 620007869, 620007870, 620007871, 620007872, 620007873, 620007874, 620007875, 620007876, 620007877, 620007878, 620007879, 620007880, 620007881, 620007882, 620007883, 620007884, 620007885, 620007886, 620007916, 620007917, 620007918, 620007919, 620007920, 620007921, 620007922, 620007923, 620007924, 620007925, 620007926, 620007927, 620007928, 620007929, 620007930, 620007931, 620007932, 620007933, 620007943, 620007944, 620007997, 620008016, 620008031, 620008034, 620008035, 620008036, 620008037, 620008040, 620008041, 620008059, 620008063, 620008064, 620008065, 620008066, 620008067, 620008068, 620008069, 620008281, 620008282, 620008283, 620008285, 620008291, 620008305, 620008345, 620008347, 620008452, 620008453, 620008454, 620008455, 620008456, 620008457, 620008458, 620008459, 620008460, 620008461, 620008462, 620008463, 620008464, 620008465, 620008466, 620008467, 620008468, 620008469, 620008470, 620008471, 620008472, 620008516, 620008517, 620008602, 620008603, 620008611, 620008652, 620008670, 620008677, 620008697, 620008701, 620008715, 620008718, 620008755, 620008781, 620009090, 620009091, 620009092, 620009093, 620009106, 620009118, 620009126, 620009127, 620009306, 620009307, 620009318, 620009319, 620009355, 620009356, 620009357, 620009358, 620009359, 620009360, 620009361, 620009362, 620009363, 620009364, 620009365, 620009366, 620009367, 620009368, 620009369, 620009370, 620009371, 620009372, 620009373, 620009374, 620009375, 620009376, 620009377, 620009378, 620009379, 620009380, 620009381, 620009382, 620009383, 620009384, 620009385, 620009386, 620009387, 620009388, 620009389, 620009390, 620009391, 620009392, 620009393, 620009394, 620009395, 620009396, 620009397, 620009400, 620009401, 620009430, 620009434, 620009442, 620009443, 620009453, 620009609, 620009610, 620252201, 620252204, 620252207, 620252208, 620252210, 620252701, 620252707, 620252712, 620252713, 620253501, 620253701, 620253703, 620253729, 620254403, 620254406, 620254407, 620254410, 620254411, 620254412, 620254413, 620254416, 620254417, 620254418, 620254419, 620254420, 620254421, 620254422, 620254424, 620254425, 620254426, 620254501, 620254509, 620254517, 620254522, 620254527, 620254532, 620254544, 620254553, 620254554, 620254560, 620255102, 620255302, 620255401, 620255403, 620255408, 620255417, 620255420, 620256101, 620256201, 620262802, 620263001, 620263201, 620263401, 620263901, 620264101, 620264701, 620266102, 620266110, 620266112, 620266119, 620266125, 620266131, 620266132, 620266138, 620266148, 620266301, 620266304, 620266305, 620266409, 620266412, 620266413, 620266901, 620267001, 620268501, 620269701, 620269703, 620269704, 620269809, 620269833, 620269834, 620270101, 620273914, 620281201, 620281304, 620281601, 620281801, 620282504, 620282601, 620289301, 620289701, 620290102, 620290104, 620290106, 620290111, 620293401, 620294901, 620295101, 620295601, 620295802, 620302701, 620307001, 620307006, 620307010, 620307012, 620307017, 620307026, 620307403, 620307601, 620307701, 620307702, 620317401, 620317603, 620318401, 620318701, 620318801, 620318901, 620318903, 620319101, 620319301, 620319704, 620319901, 620320201, 620320301, 620320401, 620320403, 620320601, 620320701, 620322401, 620323403, 620326701, 620326801, 620327001, 620327202, 620327602, 620328101, 620328201, 620328401, 620328602, 620329002, 620347801, 620348001, 620348401, 620348801, 620349001, 620349101, 620349302, 620349501, 620349901, 620350201, 621243302, 621243304, 621243401, 621243402, 621243501, 621243601, 621243701, 621243801, 621244101, 621244202, 621244402, 621244404, 621244501, 621244502, 621244602, 621244702, 621244801, 621245002, 621245201, 621245301, 621245402, 621245501, 621245602, 621245801, 621245901, 621246201, 621246301, 621246304, 621246401, 621246501, 621248601, 621248701, 621248801, 621248803, 621248901, 621249001, 621249102, 621249103, 621252701, 621252901, 621253102, 621253301, 621253401, 621253602, 621256201, 621365602, 621365713, 621365728, 621365736, 621365810, 621365817, 621365821, 621365822, 621365823, 621366703, 621366706, 621366715, 621366718, 621366720, 621399302, 621399401, 621399501, 621399602, 621399902, 621400001, 621400301, 621400302, 621400401, 621400502, 621400601, 621400901, 621401201, 621401303, 621401401, 621401501, 621401603, 621401701, 621402202, 621402601, 621402701, 621403301, 621468802, 621469201, 621469301, 621469401, 621469501, 621469601, 621469801, 621469901, 621470001, 621470101, 621470201, 621470301, 621470401, 621470601, 621470703, 621470902, 621471001, 621471103, 621520501, 621520504, 621520601, 621520604, 621525601, 621526102, 621526202, 621526301, 621526601, 621526703, 621526803, 621530201, 621530501, 621573401, 621573402, 621573601, 621573701, 621575701, 621575702, 621579602, 621621501, 621621601, 621622501, 621622701, 621622801, 621622901, 621623001, 621623101, 621628601, 621628701, 621629301, 621630801, 621630901, 621631501, 621631601, 621631801, 621631901, 621632201, 621632301, 621632401, 621632501, 621632804, 621632904, 621633801, 621634701, 621634801, 621635101, 621635901, 621636001, 621636401, 621636501, 621636601, 621636701, 621637101, 621638003, 621638004, 621638103, 621638104, 621638801, 621638901, 621640901, 621641001, 621641101, 621641201, 621641601, 621641701, 621641801, 621641901, 621642802, 621642902, 621671702, 621671802, 621675601, 621675701, 621676601, 621681701, 621681801, 621681901, 621682001, 621682801, 621682901, 621683601, 621684301, 621684401, 621685001, 621685101, 621685201, 621685401, 621689701, 621689801, 621720801, 621726901, 621726903, 621727001, 621727003, 621730802, 621730902, 621733201, 621736902, 621739203, 621739403, 621739601, 621739702, 621744701, 621744801, 621749801, 621750401, 621751001, 621751201, 621751301, 621769401, 621769402, 621770001, 621783103, 621793502, 621793602, 621835001, 621835101, 621835201, 621838103, 621838203, 621838303, 621846902, 621847002, 621847203, 621847303, 621852302, 621852402, 621852502, 621852602, 621852702, 621853103, 621853203, 621855203, 621857103, 621865301, 621866501, 621867101, 621890901, 621891001, 621918601, 621918701, 621926601, 621926701, 621931301, 621931401, 621931901, 621932001, 621932501, 621932701, 621932801, 621932901, 621933001, 621934401, 621934402, 621934403, 621934501, 621934502, 621934503, 621934601, 621934701, 621936101, 621936201, 621936801, 621936803, 621936901, 621936903, 621937301, 621937401, 621938301, 621938302, 621938501, 621938601, 621941201, 621941301, 621941401, 621941402, 621941501, 621941502, 621941601, 621941701, 621942301, 621942401, 621942501, 621944301, 621944401, 621945101, 621945201, 621945301, 621945401, 621945501, 621945601, 621945602, 621945701, 621945702, 621948101, 621948102, 621948401, 621948501, 621949101, 621949201, 621951201, 621951301, 621952601, 621952603, 621952605, 621952701, 621952703, 621952705, 621953901, 621954601, 621954603, 621954701, 621954703, 621956401, 621956402, 621956403, 621956801, 621956901, 621957301, 621957401, 621958901, 621959001, 621959101, 621959201, 621959301, 621959401, 621959501, 621959601, 621959701, 621959801, 621960401, 621960402, 621960601, 621960602, 621960701, 621960702, 621963001, 621963101, 621963201, 621963901, 621963902, 621965001, 621965002, 621965401, 621965402, 621966001, 621966002, 621966101, 621966102, 621966201, 621966202, 621966301, 621966302, 621973001, 621973101, 621974401, 621974402, 621974501, 621974601, 621975301, 621975302, 621977100, 621977101, 621977601, 621977602, 621977604, 621978501, 621978502, 621978701, 621978801, 621979801, 621979802, 621980801, 621982301, 621982801, 621982803, 621982805, 621982901, 621982903, 621982905, 621985101, 621985102, 621985201, 621985202, 621985501, 621985601, 621986501, 621986601, 621987001, 621987002, 621990001, 621990101, 621991101, 621991201, 621991501, 621991601, 621991701, 621991702, 621992201, 621992501, 621992502, 621992601, 621992602, 621999101, 622002701, 622002801, 622006801, 622010101, 622018401, 622018501, 622022201, 622022301, 622023701, 622023901, 622024201, 622024301, 622025401, 622025404, 622025501, 622025504, 622026801, 622028801, 622028802, 622029401, 622029402, 622029501, 622029502, 622029503, 622029601, 622029602, 622031901, 622031902, 622035901, 622035902, 622038701, 622038702, 622039201, 622039202, 622039301, 622039302, 622039401, 622039402, 622047001, 622047002, 622049001, 622049101, 622049501, 622049601, 622049701, 622049801, 622050101, 622050201, 622050301, 622052101, 622052102, 622052401, 622052402, 622054101, 622054201, 622054202, 622055501, 622055502, 622057901, 622058601, 622058602, 622058603, 622060401, 622060402, 622060701, 622060702, 622062801, 622062802, 622063901, 622063902, 622064001, 622064002, 622064201, 622064202, 622064301, 622064302, 622064401, 622064403, 622064501, 622064502, 622064601, 622064602, 622064801, 622064802, 622066901, 622067201, 622067202, 622068001, 622068002, 622071301, 622071401, 622072601, 622076001, 622076101, 622076901, 622076902, 622077001, 622077002, 622077601, 622077701, 622077702, 622080301, 622080302, 622082701, 622082702, 622082801, 622082802, 622095501, 622095601, 622096301, 622096302, 622097101, 622098601, 622098602, 622098701, 622098702, 622099401, 622099501, 622100201, 622100202, 622100301, 622100302, 622101901, 622102001, 622102101, 622102201, 622102203, 622102301, 622102303, 622105701, 622106801, 622106802, 622108001, 622108002, 622108801, 622108802, 622109301, 622110101, 622110102, 622110201, 622110202, 622110301, 622117001, 622117002, 622117101, 622117102, 622117301, 622117401, 622123501, 622123502, 622125301, 622125302, 622125401, 622125402, 622126201, 622126301, 622128401, 622128402, 622128501, 622128502, 622134301, 622134401, 622134501, 622136701, 622136801, 622136901, 622137601, 622137801, 622137901, 622138001, 622138901, 622139001, 622139101, 622140101, 622140201, 622140301, 622141201, 622142401, 622142402, 622142501, 622142502, 622142601, 622142701, 622142801, 622143001, 622143101, 622143501, 622143601, 622144801, 622144901, 622145001, 622145901, 622146001, 622146101, 622146401, 622146403, 622146501, 622146502, 622146601, 622146603, 622146801, 622146802, 622146901, 622146903, 622147001, 622147002, 622150101, 622150201, 622150301, 622151701, 622151801, 622151901, 622152401, 622152501, 622152601, 622153001, 622153101, 622153201, 622153501, 622153601, 622153701, 622155001, 622155002, 622155101, 622155102, 622155201, 622155202, 622156601, 622156701, 622156801, 622158001, 622158101, 622158201, 622160301, 622160401, 622160501, 622161601, 622161602, 622161701, 622161702, 622162201, 622162301, 622162401, 622163001, 622163101, 622163201, 622163901, 622165301, 622165401, 622165501, 622166501, 622166601, 622166701, 622168601, 622168701, 622168801, 622171001, 622171101, 622171201, 622172301, 622172401, 622172501, 622173501, 622173601, 622173701, 622174601, 622174701, 622174801, 622176001, 622176101, 622176201, 622177001, 622177101, 622177201, 622179501, 622180801, 622181201, 622181301, 622181401, 622181901, 622182001, 622182101, 622183801, 622184701, 622187801, 622187901, 622189801, 622189901, 622193201, 622198101, 622199201, 622199301, 622201301, 622201401, 622202301, 622202901, 622206801, 622206803, 622206901, 622206903, 622207401, 622207501, 622208801, 622211901, 622213801, 622213802, 622214101, 622214201, 622214301, 622215001, 622216201, 622216301, 622219501, 622219601, 622221101, 622221501, 622224601, 622230401, 622230501, 622233401, 622233501, 622233601, 622234501, 622236201, 622236301, 622239501, 622240001, 622240002, 622240101, 622240102, 622241101, 622241201, 622242401, 622243401, 622243501, 622243801, 622247001, 622247101, 622248901, 622249501, 622249601, 622249701, 622249801, 622251401, 622251501, 622253701, 622253801, 622253901, 622255401, 622255501, 622255601, 622255701, 622257701, 622257801, 622257901, 622258201, 622258301, 622260701, 622260901, 622261701, 622264101, 622264201, 622267201, 622267301, 622267901, 622269701, 622271001, 622272201, 622272301, 622272401, 622273501, 622279501, 622280101, 622282601, 622284001, 622284002, 622284101, 622284601, 622284801, 622285601, 622287301, 622288501, 622289401, 622289402, 622290901, 622296701, 622296801, 622298501, 622301601, 622307501, 622307601, 622307701, 622307801, 622308601, 622308701, 622308801, 622308901, 622309101, 622309600, 622309700, 622309800, 622309900, 622310000, 622310500, 622310600, 622312300, 622312500, 622312800, 622314900, 622315000, 622315100, 622315800, 622318700, 622318800, 622318900, 622319100, 622319700, 622319800, 622320100, 622320200, 622320400, 622320500, 622320600, 622320700, 622321100, 622321800, 622322800, 622323000, 622323100, 622323300, 622323400, 622323500, 622324500, 622324800, 622324900, 622325000, 622325700, 622333601, 622333602, 622333701, 622333702, 622333801, 622333802, 622333901, 622333902, 622334001, 622334002, 622334301, 622334401, 622334501, 622334601, 622334701, 622334901, 622335001, 622335101, 622335201, 622335501, 622336901, 622337001, 622337101, 622337401, 622337501, 622337601, 622337701, 622337901, 622338801, 622338901, 622339001, 622339101, 622339201, 622339301, 622339401, 622340401, 622340402, 622340501, 622340502, 622340601, 622340602, 622340701, 622340702, 622340801, 622341001, 622341101, 622341201, 622341301, 622341401, 622341701, 622341801, 622342101, 622342201, 622342301, 622342401, 622342501, 622343001, 622343101, 622343201, 622343301, 622343401, 622343501, 622343601, 622343602, 622343701, 622343702, 622343801, 622343802, 622343901, 622343902, 622344401, 622344403, 622345701, 622345801, 622345901, 622346001, 622346101, 622346401, 622346501, 622346601, 622346701, 622346801, 622347301, 622347501, 622347601, 622347701, 622347801, 622348001, 622348101, 622348201, 622348301, 622348401, 622348601, 622348801, 622348901, 622349001, 622349101, 622349301, 622349801, 622349901, 622350001, 622350101, 622350801, 622350901, 622351001, 622351101, 622351201, 622351501, 622351502, 622351601, 622351602, 622351701, 622351702, 622351801, 622351802, 622351901, 622352401, 622352501, 622352601, 622352701, 622352801, 622353201, 622353301, 622353401, 622353501, 622353601, 622353701, 622353801, 622353901, 622354001, 622354101, 622354201, 622354501, 622355001, 622355101, 622355201, 622355301, 622355501, 622355601, 622355701, 622355801, 622355901, 622356001, 622356101, 622356701, 622356801, 622356901, 622357001, 622357101, 622357201, 622357301, 622357401, 622357501, 622357801, 622357901, 622358001, 622358101, 622358601, 622358701, 622358801, 622358901, 622359001, 622359401, 622359501, 622359601, 622359701, 622359801, 622360701, 622360801, 622360901, 622361501, 622361601, 622361701, 622361801, 622361901, 622362001, 622362101, 622362201, 622362301, 622362401, 622362501, 622362601, 622362801, 622362901, 622363001, 622365401, 622365501, 622365601, 622365701, 622367401, 622367501, 622367601, 622367701, 622368401, 622368501, 622368601, 622368701, 622369201, 622369301, 622369401, 622369501, 622369601, 622369701, 622369801, 622369901, 622370401, 622370501, 622370601, 622370701, 622371301, 622371401, 622371501, 622371601, 622372001, 622372101, 622372201, 622372301, 622373401, 622373501, 622373601, 622373701, 622376501, 622376601, 622376701, 622376801, 622377001, 622377101, 622377201, 622377301, 622377401, 622377501, 622377601, 622377701, 622378401, 622378501, 622378601, 622378701, 622379601, 622379701, 622379801, 622379901, 622380501, 622380601, 622380701, 622380801, 622380901, 622381001, 622381501, 622381601, 622381701, 622381801, 622382201, 622382301, 622382401, 622382501, 622382601, 622382701, 622382801, 622382901, 622383401, 622383701, 622383801, 622383901, 622384001, 622385001, 622385003, 622385101, 622385103, 622385201, 622385301, 622385401, 622385501, 622385601, 622386001, 622386401, 622386501, 622386601, 622386701, 622387101, 622387201, 622388301, 622388401, 622389301, 622390201, 622390301, 622390401, 622390501, 622390601, 622390701, 622390801, 622390901, 622391301, 622391401, 622391501, 622391601, 622392601, 622392701, 622392801, 622392901, 622393401, 622393501, 622393601, 622393701, 622394201, 622394203, 622394301, 622394303, 622395401, 622395501, 622395601, 622395701, 622396101, 622396201, 622396301, 622396401, 622397701, 622397801, 622397901, 622398001, 622398101, 622398201, 622398301, 622398401, 622398501, 622398601, 622398701, 622398801, 622398901, 622399001, 622399101, 622399201, 622399301, 622399401, 622399501, 622399601, 622399701, 622399801, 622399901, 622400001, 622412101, 622412201, 622412301, 622412401, 622422901, 622439701, 622443001, 622443101, 622443501, 622443901, 622444001, 622444101, 622444201, 622444301, 622444401, 622444501, 622445301, 622445401, 622445501, 622445601, 622445701, 622446601, 622447601, 622447701, 622447801, 622447901, 622448001, 622451101, 622451501, 622451801, 622451901, 622452001, 622452101, 622452401, 622452501, 622452601, 622452701, 622452801, 622452901, 622453001, 622453101, 622453201, 622453301, 622453401, 622454201, 622454301, 622454801, 622455101, 622455501, 622455502, 622456301, 622456501, 622456601, 622456701, 622456801, 622456901, 622457001, 622457101, 622457201, 622457301, 622458401, 622459101, 622459201, 622459301, 622459401, 622459501, 622460101, 622460201, 622460301, 622461101, 622461201, 622461301, 622461401, 622462701, 622462801, 622462901, 622463001, 622463101, 622463201, 622463801, 622464201, 622464301, 622465201, 622465301, 622465401, 622465501, 622465601, 622465701, 622465801, 622465901, 622466001, 622467301, 622467401, 622467801, 622467901, 622468001, 622468101, 622468201, 622468301, 622468401, 622468801, 622468901, 622469401, 622469501, 622469601, 622469701, 622472701, 622473900, 622474000, 622474100, 622474200, 622474300, 622474400, 622474500, 622474600, 622474700, 622474800, 622474900, 622479201, 622479301, 622479801, 622480901, 622481001, 622481401, 622481501, 622481801, 622482101, 622483301, 622486501, 622486601, 622489101, 622489102, 622490001, 622490801, 622490901, 622491201, 622493201, 622493501, 622494301, 622494302, 622494401, 622494403, 622494601, 622495601, 622495701, 622495801, 622495901, 622496101, 622496103, 622496201, 622496203, 622496301, 622496501, 622496601, 622496701, 622499901, 622500301, 622500401, 622501601, 622501701, 622502801, 622504201, 622504301, 622504501, 622504601, 622504901, 622505801, 622506001, 622506801, 622506901, 622507201, 622511501, 622512601, 622512603, 622513501, 622513601, 622515001, 622515101, 622516502, 622519601, 622519701, 622519901, 622520001, 622520101, 622520501, 622524101, 622524201, 622524301, 622524401, 622527001, 622527101, 622527201, 622527301, 622529101, 622529201, 622531601, 622531701, 622535101, 622535201, 622535301, 622535401, 622535501, 622535601, 622535701, 622536101, 622536201, 622536301, 622537001, 622537101, 622537201, 622540001, 622540501, 622540601, 622540701, 622540901, 622541001, 622541101, 622542801, 622543001, 622543101, 622543301, 622543401, 622543501, 622543601, 622543701, 622543801, 622543901, 622544001, 622544101, 622546001, 622546101, 622546201, 622546501, 622547101, 622547201, 622547301, 622547302, 622547401, 622547402, 622547501, 622547701, 622548901, 622548902, 622549301, 622549401, 622549501, 622551501, 622551601, 622551701, 622553401, 622553501, 622553601, 622557601, 622557701, 622557801, 622558101, 622558201, 622558301, 622558401, 622558501, 622558601, 622558701, 622558801, 622558901, 622559501, 622559601, 622559701, 622562801, 622562901, 622563001, 622563601, 622563701, 622563801, 622563901, 622564001, 622564101, 622565101, 622565201, 622565301, 622566601, 622566701, 622566801, 622566901, 622567001, 622567401, 622568801, 622568901, 622569001, 622569101, 622569201, 622569301, 622569401, 622570301, 622570401, 622570501, 622570601, 622570701, 622571101, 622571201, 622571301, 622571401, 622571501, 622571601, 622571701, 622574701, 622574801, 622574901, 622575001, 622577201, 622577301, 622577401, 622577501, 622577601, 622577701, 622577801, 622579401, 622579501, 622579601, 622579701, 622579801, 622579901, 622580001, 622580101, 622580201, 622580301, 622582901, 622583001, 622583101, 622584301, 622584303, 622584401, 622584403, 622584501, 622584503, 622584601, 622584603, 622585601, 622585701, 622585801, 622585901, 622586801, 622586901, 622587001, 622587501, 622587601, 622587701, 622587801, 622587901, 622588001, 622588101, 622589401, 622589501, 622589601, 622589701, 622591001, 622591101, 622591201, 622591301, 622591401, 622591501, 622591601, 622592101, 622592201, 622592301, 622592401, 622593301, 622593401, 622593501, 622593601, 622593701, 622593801, 622593901, 622594001, 622594101, 622594201, 622594301, 622594901, 622595001, 622595101, 622595201, 622597001, 622597101, 622597201, 622597301, 622597601, 622597701, 622597801, 622597901, 622598001, 622598101, 622598201, 622598701, 622598801, 622598901, 622599601, 622599701, 622599801, 622599901, 622600001, 622600101, 622600201, 622600401, 622600501, 622601601, 622601701, 622601801, 622601901, 622602001, 622602101, 622602201, 622602301, 622602401, 622602501, 622602601, 622602701, 622602801, 622602901, 622603001, 622603101, 622603201, 622603301, 622603401, 622603501, 622603601, 622603701, 622612000, 622612100, 622613400, 622613500, 622613600, 622613700, 622613800, 622613900, 622614000, 622614100, 622615100, 622615200, 622615300, 622615400, 622617500, 622617600, 622620101, 622620201, 622620301, 622620401, 622620501, 622622701, 622622801, 622625201, 622625301, 622625901, 622626701, 622626801, 622627501, 622627601, 622629301, 622631501, 622631601, 622631701, 622631801, 622631901, 622632001, 622632101, 622632301, 622632401, 622632601, 622632603, 622632701, 622632703, 622633601, 622633701, 622633801, 622633901, 622634001, 622635301, 622635401, 622635501, 622635601, 622636701, 622636801, 622636901, 622637001, 622637501, 622637601, 622639701, 622639801, 622641401, 622641501, 622641601, 622642501, 622642601, 622644401, 622644501, 622644601, 622644701, 622652101, 622663701, 622663801, 622663901, 622664801, 622664901, 622665001, 622669401, 622670101, 622670201, 622670301, 622689200, 622689300, 622689400, 622689500, 622689600, 622689700, 622690100, 622690200, 622690300, 622690400, 622690500, 622690600, 622690700, 622690800, 622691300, 622691600, 622718800, 622718900, 622719000, 622719100, 622719200, 622719300, 622721500, 622721600, 622721700, 622721800, 622721900, 622722000, 622722100, 622722200, 622722300, 622722400, 622722500, 622722600, 622722700, 622722800, 622722900, 622723000, 622723100, 622723200, 622723300, 622723400, 622723500, 622723600, 622723700, 622723800, 622723900, 622724000, 622724100, 622724200, 622724300, 622724400, 622724500, 622724600, 622724700, 622724800, 622724900, 622725000, 622725100, 622725200, 622725300, 622725400, 622725500, 622725600, 622725700, 622726000, 622726100, 622726200, 622726300, 622727100, 622727200, 622727300, 622727400, 622727500, 622727600, 622727700, 622727800, 622728100, 622728200, 622728300, 622820901, 622821001, 622821101, 622833901, 622834001, 622834101, 622834201, 622834301, 622834501, 622834601, 622834701, 622834801, 622844001, 622844101, 622844901, 622845001, 622846200, 622846300, 622850701, 622850801, 622852101, 622852201, 622857601, 622857701, 622858801, 622858901, 622859001, 622859101, 622865601, 622865701, 622866201, 622866301, 622867801, 622867901, 622872701, 622872801, 622872901, 622873001, 622875901, 622876001, 622876101, 622876501, 622876601, 622876701, 622876801, 622876901, 622880301, 622880401, 622890300, 622890600, 622890700, 622890800, 622890900, 622891000, 622891100, 622891200, 622891300, 622891400, 622891500, 622891600, 622891700, 622891800, 622891900, 622892000, 622892200, 622892300, 622893000, 622893100

Drug for Dyslipidemia

610406035, 610406108, 610406254, 610406405, 610407028, 610407029, 610421331, 610421332, 610421333, 610421343, 610421344, 610421345, 610422004, 610422018, 610422023, 610422030, 610422031, 610422038, 610422052, 610422053, 610422054, 610422055, 610422056, 610422057, 610422059, 610422062, 610422098, 610422120, 610422123, 610422206, 610422221, 610422222, 610422232, 610422257, 610422258, 610422259, 610422261, 610422262, 610422263, 610422264, 610422265, 610422276, 610422286, 610422290, 610422292, 610422305, 610432002, 610432003, 610432017, 610432018, 610433003, 610433008, 610433045, 610433091, 610433105, 610433109, 610433110, 610433123, 610433125, 610433126, 610433127, 610433128, 610433130, 610433131, 610433143, 610443013, 610443014, 610444136, 610454063, 610454084, 610454085, 610461121, 610462007, 610462015, 610462016, 610463087, 610463151, 610463152, 610470012, 610470013, 610470014, 612180002, 612180003, 612180004, 612180006, 612180011, 612180028, 612180029, 612180032, 612180035, 612180042, 612180055, 612180056, 612180067, 612180070, 612180099, 612180106, 612180111, 612180120, 612180124, 612180135, 612180140, 612180141, 612180183, 612180184, 612180185, 612180186, 612180193, 612180194, 612180198, 612180200, 612180201, 612180202, 612180203, 612180206, 612180208, 612180212, 612180213, 612180214, 612180215, 612180216, 612180218, 612180231, 612180232, 612180233, 612180244, 612180247, 612180248, 612180259, 612180260, 612180262, 612180263, 612180264, 612180265, 612180266, 612180267, 612180268, 612180270, 612180271, 612180272, 612180273, 612180274, 612180276, 612180277, 612180278, 612180280, 612180282, 612180283, 612180284, 612180285, 612180286, 612180287, 612180288, 612180289, 612180290, 612180291, 612180292, 612190001, 612190002, 612190067, 612190069, 612190079, 612190124, 612190160, 612190161, 612190172, 612190177, 612190186, 612190187, 612190258, 612900003, 612900006, 612900012, 612900013, 612900020, 612900021, 612900022, 612900041, 612900042, 612900044, 612900049, 612900068, 612900071, 612900072, 612900110, 612900113, 612900114, 612900115, 612900116, 612900118, 613130058, 613130135, 613130165, 613130166, 613130178, 613130179, 613130185, 613130187, 613130188, 613130189, 613130190, 613130192, 613130204, 613130219, 613130220, 613130221, 613130222, 613130223, 613130238, 613130239, 613130240, 613130245, 613130315, 613130365, 613130366, 613130367, 613130379, 613130380, 613130394, 613130395, 613130462, 613130503, 613130504, 613130547, 613390006, 620000013, 620000014, 620000025, 620000026, 620000038, 620000039, 620000051, 620000052, 620000053, 620000054, 620000055, 620000070, 620000071, 620000103, 620000104, 620000105, 620000106, 620000107, 620000108, 620000109, 620000110, 620000111, 620000112, 620000113, 620000114, 620000115, 620000116, 620000140, 620000141, 620000145, 620000146, 620000149, 620000150, 620000151, 620000152, 620000153, 620000154, 620000155, 620000156, 620000157, 620000158, 620000159, 620000160, 620000161, 620000162, 620000163, 620000164, 620000169, 620000171, 620000174, 620000175, 620000176, 620000177, 620000178, 620000179, 620000422, 620000423, 620001883, 620001902, 620001924, 620002049, 620002050, 620002051, 620002052, 620002093, 620002094, 620002108, 620002109, 620002117, 620002118, 620002123, 620002162, 620002163, 620002164, 620002165, 620002166, 620002423, 620002424, 620002433, 620002434, 620002477, 620002478, 620002508, 620002540, 620002541, 620002546, 620002735, 620002736, 620002798, 620002799, 620002800, 620002801, 620002802, 620002879, 620002880, 620003595, 620003596, 620003597, 620003623, 620003668, 620003669, 620004037, 620004038, 620004085, 620004459, 620004519, 620004546, 620004547, 620004607, 620004608, 620004609, 620004868, 620004942, 620004943, 620004985, 620005032, 620005064, 620005513, 620005785, 620005915, 620005920, 620005931, 620005932, 620005938, 620005965, 620006039, 620006070, 620006072, 620006102, 620006108, 620006115, 620006164, 620006574, 620006601, 620006681, 620006858, 620006870, 620006904, 620006936, 620007887, 620007888, 620007889, 620007890, 620007891, 620007892, 620007893, 620007894, 620007895, 620007896, 620007897, 620007898, 620007993, 620007994, 620007995, 620008053, 620008054, 620008055, 620008056, 620008111, 620008112, 620008113, 620008294, 620008508, 620008610, 620008631, 620008669, 620008679, 620008684, 620008685, 620008686, 620008706, 620008710, 620008761, 620008766, 620008767, 620008784, 620009208, 620009218, 620009322, 620009323, 620009324, 620009325, 620009425, 620009426, 620338305, 620338317, 620339201, 620339401, 620339501, 620339603, 620340201, 620340602, 620340603, 620340901, 620341001, 620341102, 620341301, 620344306, 620346001, 620346008, 620346018, 620346023, 620346029, 620346101, 620352901, 620353101, 620353201, 620353202, 620353503, 620353601, 620353804, 620672913, 620672914, 620672920, 620673501, 620673513, 620673518, 620689802, 620690301, 620690701, 620691002, 620691102, 620695916, 620696210, 620696601, 620697001, 620697701, 620698003, 620815302, 620815601, 620815903, 620816201, 620816301, 620816501, 620816805, 620817001, 620817103, 620817504, 620817702, 621254601, 621315603, 621521301, 621521401, 621523101, 621523201, 621524102, 621524402, 621524504, 621525701, 621525801, 621528401, 621528501, 621528602, 621528702, 621528801, 621528901, 621529001, 621529101, 621531001, 621531101, 621531703, 621532401, 621532501, 621532601, 621532902, 621533002, 621533101, 621533201, 621533501, 621533601, 621533801, 621533901, 621534003, 621534101, 621534204, 621534301, 621623603, 621635202, 621635301, 621635403, 621635504, 621639001, 621639101, 621639701, 621639801, 621643301, 621643401, 621643501, 621643601, 621675101, 621694001, 621752501, 621776502, 621869505, 621869605, 621869705, 621932701, 621932801, 621932901, 621933001, 621934801, 621934901, 621935001, 621948701, 621948801, 621955001, 621955003, 621955101, 621959901, 621959902, 621960001, 621960002, 621960101, 621960102, 621964101, 621964201, 621964301, 621964401, 621964501, 621964601, 621981401, 621981403, 622015101, 622015201, 622015301, 622026701, 622026702, 622039501, 622039601, 622052801, 622055601, 622055602, 622062701, 622071601, 622075801, 622075901, 622076401, 622076501, 622090701, 622090801, 622096101, 622096102, 622096801, 622096901, 622098401, 622098501, 622099101, 622099201, 622102501, 622102502, 622107601, 622107701, 622108301, 622110401, 622110501, 622116801, 622116802, 622116901, 622116902, 622126901, 622127001, 622128201, 622128301, 622136401, 622139600, 622143801, 622143901, 622152001, 622152101, 622161801, 622161901, 622165601, 622165701, 622167601, 622167701, 622169901, 622169902, 622170001, 622170002, 622170101, 622170201, 622180601, 622180602, 622180701, 622180702, 622186601, 622186701, 622187601, 622187701, 622198801, 622204801, 622204901, 622217101, 622217201, 622223601, 622239201, 622239301, 622241301, 622241401, 622244801, 622244901, 622252001, 622252101, 622268001, 622268101, 622268201, 622269101, 622269201, 622270001, 622270101, 622271801, 622271901, 622273101, 622273103, 622273201, 622273203, 622273301, 622273303, 622274901, 622275001, 622275101, 622276301, 622276401, 622276501, 622280201, 622280301, 622280401, 622280501, 622280601, 622280701, 622280801, 622282201, 622282301, 622283701, 622283801, 622285001, 622285002, 622285101, 622285102, 622286201, 622286301, 622286401, 622287601, 622289501, 622289601, 622291801, 622291901, 622292001, 622292301, 622292401, 622292501, 622293301, 622293401, 622294301, 622294401, 622294501, 622296001, 622296101, 622296201, 622297101, 622297201, 622298001, 622298101, 622298201, 622299001, 622299101, 622302401, 622302501, 622302801, 622302901, 622304601, 622304701, 622304801, 622304901, 622315400, 622315500, 622315600, 622321900, 622322700, 622322900, 622342801, 622347401, 622347402, 622359101, 622360101, 622360201, 622362701, 622365801, 622372401, 622387001, 622387601, 622392501, 622406901, 622419701, 622419801, 622419901, 622421601, 622421701, 622421801, 622426201, 622427701, 622427801, 622431401, 622434301, 622434401, 622441101, 622441201, 622444001, 622444101, 622444201, 622444301, 622445401, 622445501, 622445601, 622445701, 622447601, 622447701, 622447801, 622447901, 622452401, 622452501, 622452601, 622452701, 622453001, 622453101, 622453201, 622453301, 622456901, 622457001, 622457101, 622457201, 622457701, 622457801, 622457901, 622461101, 622461201, 622461301, 622461401, 622462701, 622462801, 622462901, 622463001, 622464901, 622465001, 622465101, 622465301, 622465401, 622465501, 622465601, 622467801, 622467901, 622468001, 622468101, 622468501, 622468601, 622468701, 622471801, 622475000, 622475100, 622488901, 622508401, 622508501, 622508601, 622508701, 622512001, 622512003, 622512101, 622512103, 622512201, 622512203, 622516701, 622516801, 622516901, 622522101, 622522201, 622522301, 622524501, 622524601, 622524701, 622528901, 622529001, 622537301, 622537401, 622568601, 622571801, 622571901, 622572801, 622572901, 622573101, 622575201, 622575301, 622575401, 622575501, 622575601, 622575701, 622577901, 622578001, 622578101, 622578201, 622578401, 622578501, 622578601, 622578701, 622578801, 622579101, 622581601, 622581701, 622581801, 622581901, 622582001, 622582101, 622582501, 622582601, 622582701, 622582801, 622584101, 622584201, 622584701, 622584703, 622584801, 622584803, 622586001, 622586101, 622586201, 622586301, 622588801, 622588901, 622589001, 622589101, 622590101, 622590201, 622590301, 622590401, 622590501, 622590601, 622591701, 622591801, 622591901, 622592001, 622592501, 622592601, 622592701, 622592801, 622592901, 622593001, 622593101, 622593201, 622595301, 622595401, 622598301, 622598401, 622598501, 622598601, 622599201, 622599301, 622599401, 622599501, 622600301, 622600601, 622600701, 622600801, 622600901, 622601201, 622601301, 622601401, 622601501, 622604001, 622604101, 622604201, 622605001, 622605101, 622605201, 622605301, 622605401, 622606601, 622606701, 622610500, 622615600, 622615700, 622615800, 622615900, 622640801, 622640901, 622644801, 622644901, 622660001, 622660101, 622665901, 622666001, 622666101, 622676701, 622676801, 622691000, 622691800, 622692400, 622692500, 622692600, 622692700, 622704001, 622704101, 622728600, 622728700, 622728800, 622728900, 622738900, 622739000, 622739100, 622784901, 622786001, 622789601, 622789701, 622790901, 622791901, 622792901, 622794301, 622795201, 622797701, 622799601, 622801101, 622803401, 622803501, 622803601, 622803701, 622805001, 622806401, 622808201, 622809701, 622812801, 622814501, 622817301, 622846700, 622886101, 622892400, 622892500, 622892600, 622892700, 622892800, 622892900, 622893000, 622893100, 622896000, 622896100, 622896200, 622896300, 622902400, 622908201, 622910601, 622913601, 622917901

List of Japanese Codes for Self-Injection Fee for Type 1 Diabetes

114009910, 114010010, 114010110, 114010210, 114010970, 114015510, 114015610, 114046110, 114050210
