# Correction: Trends in Diabetic Medication Use and Hypoglycemia Incidence Among Older Adults in Japan: A Retrospective Observational Study and a 10-Year Analysis Based on Level of Care Need Using Linked Medical and Long-Term Care Data

**DOI:** 10.7759/cureus.c252

**Published:** 2025-08-20

**Authors:** Yasuhiro Niida, Yuichi Nishioka, Tomoya Myojin, Tatsuya Noda, Yutaka Takahashi, Tomoaki Imamura

**Affiliations:** 1 Department of Public Health, Health Management and Policy, Nara Medical University, Kashihara, JPN; 2 Department of Diabetes and Endocrinology, Nara Medical University, Kashihara, JPN

This article’s reference list has been corrected due to originally being published in the incorrect order. References 7-13 have been switched with references 11-17 and references 14-17 have been switched with references 7-10.

